# Volatilomic Signatures of AGS and SNU-1 Gastric Cancer Cell Lines

**DOI:** 10.3390/molecules27134012

**Published:** 2022-06-22

**Authors:** Daria Ślefarska-Wolak, Christine Heinzle, Andreas Leiherer, Clemens Ager, Axel Muendlein, Linda Mezmale, Marcis Leja, Alejandro H. Corvalan, Heinz Drexel, Agnieszka Królicka, Gidi Shani, Christopher A. Mayhew, Hossam Haick, Paweł Mochalski

**Affiliations:** 1 Institute for Breath Research, University of Innsbruck, 6850 Dornbirn, Austria; daria.slefarska@uibk.ac.at (D.Ś.-W.); clemens.ager@a1.net (C.A.); christopher.mayhew@uibk.ac.at (C.A.M.); 2 Institute of Chemistry, Jan Kochanowski University, 25406 Kielce, Poland; 3 Vorarlberg Institute for Vascular Investigation and Treatment (VIVIT), 6800 Feldkirch, Austria; christine.heinzle@vivit.at (C.H.); andreas.leiherer@vivit.at (A.L.); axel.muendlein@vivit.at (A.M.); heinz.drexel@extern.insel.ch (H.D.); 4 Medical Central Laboratories, 6800 Feldkirch, Austria; 5 Private University in the Principality of Liechtenstein, 9495 Triesen, Liechtenstein; 6 Institute of Clinical and Preventive Medicine, Faculty of Medicine, University of Latvia, 1586 Riga, Latvia; linda.mezmale@lu.lv (L.M.); marcis.leja@lu.lv (M.L.); 7 Riga East University Hospital, 1586 Riga, Latvia; 8 Digestive Diseases Centre GASTRO, 1586 Riga, Latvia; 9 Advanced Center for Chronic Diseases (ACCDiS), Pontificia Universidad Catolica de Chile, Santiago 833003, Chile; acorvalan@uc.cl; 10 Department of Medicine and Cardiology, Academic Teaching Hospital Bregenz, 6900 Bregenz, Austria; 11 College of Medicine, Drexel University, Philadelphia, PA 19129, USA; 12 Department of Building Materials Technology, Faculty of Materials Science and Ceramics, AGH University of Science and Technology, 30059 Krakow, Poland; krolicka@agh.edu.pl; 13 Department of Chemical Engineering, Russel Berrie Nanotechnology Institute, Technicon—Israel Institute of Technology, Haifa 3200003, Israel; gidishani@gmail.com (G.S.); hhossam@technion.ac.il (H.H.); 14 Tiroler Krebsforschungsinstitut (TKFI), 6020 Innsbruck, Austria

**Keywords:** volatilome, volatile organic compounds, gastric cancer, GC-MS, chemical footprint, SNU-1, AGS, GES-1

## Abstract

In vitro studies can help reveal the biochemical pathways underlying the origin of volatile indicators of numerous diseases. The key objective of this study is to identify the potential biomarkers of gastric cancer. For this purpose, the volatilomic signatures of two human gastric cancer cell lines, AGS (human gastric adenocarcinoma) and SNU-1 (human gastric carcinoma), and one normal gastric mucosa cell line (GES-1) were investigated. More specifically, gas chromatography mass spectrometry has been applied to pinpoint changes in cell metabolism triggered by cancer. In total, ten volatiles were found to be metabolized, and thirty-five were produced by cells under study. The volatiles consumed were mainly six aldehydes and two heterocyclics, whereas the volatiles released embraced twelve ketones, eight alcohols, six hydrocarbons, three esters, three ethers, and three aromatic compounds. The SNU-1 cell line was found to have significantly altered metabolism in comparison to normal GES-1 cells. This was manifested by the decreased production of alcohols and ketones and the upregulated emission of esters. The AGS cells exhibited the increased production of methyl ketones containing an odd number of carbons, namely 2-tridecanone, 2-pentadecanone, and 2-heptadecanone. This study provides evidence that the cancer state modifies the volatilome of human cells.

## 1. Introduction

Over the last few decades, studies on volatiles emitted or released by the human body have provided a “proof-of-principle” that the volatilome is a potentially powerful tool capable of providing novel biomarkers for medical diagnosis and therapy monitoring [1]. The volatilome is defined as a subset of the metabolome, embracing volatile organic compounds (VOCs) of various origins within the human organism. These VOCs can be the end- or by-products of vital metabolic and biochemical processes occurring in the body, or they can stem from exogenous sources such as environmental exposure, diet, drugs, or microbiota activity. They form specific biochemical signatures that contain information on various normal and abnormal processes occurring in the body.

The VOC signatures express rapid changes when diverse pathological processes occur and alter the body’s biochemistry. This includes processes such as oxidative stress, changes in enzyme activity, carbohydrate metabolism, lipid metabolism, modifications of proteins, or activation of genes. Thus, the analysis of volatile patterns coming from different bodily excretions, such as breath, urine, saliva, or sweat, provides a unique opportunity to detect these changes and thereby monitor or screen for various diseases, including cancer.

The unique feature of the volatilomic concept is that the fast and reliable information on the processes in the human organism is obtained non-invasively via analysis of volatiles emitted or secreted by the human body into its surrounding environment. Breath analysis holds, in this context, a distinguished status. This is because exhaled breath can be obtained rapidly, as often as deemed, and analyzed using simple to use and cheap analyzers based on miniature chemical sensors [2,3]. However, the volatilomic approach faces several constraints, which limit its application within a clinical setting. The main challenge here is an insufficient understanding of the metabolic origin of many volatile biomarkers in the human organism.

Gastric cancer is the fifth most common cancer and the third leading cause of cancer-related death worldwide [4]. Owing to the lack of specific symptoms in its early stage, the diagnosis of gastric cancer is often very difficult. Since an early diagnosis is crucial for a patient’s survival, a rapid and non-invasive screening system is of highest importance.

The volatilomic approach has been used extensively to investigate gastric cancer, with a major goal to provide non-invasive methods to detect and classify the cancer. In different geographic locations, a number of studies involving various e-noses and human breath have yielded promising results. Reports of e-nose breath analyzers combined with pattern recognition methods have demonstrated the potential of being able to discriminate gastric cancer patients from other cancer patients and from healthy controls, with a sensitivity and specificity ranging from 67% to 100% and from 71% to 98%, respectively [5]. A particularly exciting example of this is a recent study reported by Nakhleh et al., They have reported the non-invasive diagnosis and classification of seventeen diseases, including gastric cancer, from exhaled breath via pattern analysis [2]. Using nanomaterial-based sensors to analyze “alveolar exhaled breath samples”, Xu et al., [6] report that they could differentiate gastric cancer from benign stomach ulcers and less severe stomach conditions with a sensitivity and a specificity of 89% and 90%, respectively. Broza et al., [7] demonstrated the potential use of e-nose sensors for identifying different diseases in a population-based cohort with a sensitivity of 100% and a specificity of 79%. More recently, by employing two portable sensor-based breath analyzers, named SniffPhone, and using linear discriminant analysis, Leja et al., [8] demonstrated a clear discrimination between patients with gastric cancer and healthy controls (area under the curve, 93.8%; sensitivity, 100%; specificity, 87.5%; and overall accuracy, 91.1%).

Although all of the sensor studies highlighted above show highly promising results for developing simple to use breath tests, a major unresolved issue is the low chemical selectivity of these devices. These instruments do not identify the VOCs but instead use a pattern that is considered specific enough to identify a particular disease. To trust such analyses, and to develop the breath tests, the individual volatiles making up these patterns must be identified, and then, potentially, the metabolic processes resulting in the production of the volatiles can be understood.

This study’s key objective is to identify the potential biomarkers of gastric cancer. These can be revealed via the analysis of VOC signatures in different bodily fluids and tissues based on highly selective techniques, such as gas chromatography-mass spectrometry (GC-MS). In vitro studies, involving cell cultures, are of particular importance in this context, as the cancer cells can be isolated from the organism and their metabolism can be studied separately, divorced from the complexity of the human body.

The work presented in this paper centers upon the volatilomic signatures of two human gastric cancer cell lines, AGS (human gastric adenocarcinoma) and SNU-1 (human gastric carcinoma), compared to that from one normal gastric mucosa cell line (GES-1). More specifically, the work’s ambition is to identify VOCs produced and consumed by the cells of interest and to pinpoint changes in cell metabolism triggered by cancer. To the best of our knowledge, this paper presents the first report of the VOC signatures for the AGS and SNU-1 cells.

## 2. Results and Discussion

### 2.1. Validation Parameters

The validation parameters of the applied analytical method are presented in Table 1. Limits of detection (LOD) were calculated using the algorithm proposed by Huber [9] and the standard deviation of five blank signals. The limit of quantification (LOQ) is defined as 3× LOD. The LOD for the volatiles falls in the range of 0.02–2 ppb. The relative standard deviations (RSDs) ranged from 7.0% up to 15%, which are adequate for the goals of this study. The linearity of the instrument response was evaluated by the analysis of the residuals. The residuals were checked for the normality using the Anderson–Darling test, and a *p*-value < 0.05 was taken as being significant to reject normality. The instrument response was linear within the concentrations of the VOCs of interest, with coefficients of determination ranging from 0.979 to 0.998. Representative chromatograms from the analysis of the headspace of cells under scrutiny are provided in Supplementary Figures S9–S12.

### 2.2. Cell Cultures

The total number of cells in the measurement flasks at the time of measurement is provided in Table 2. Live–dead staining revealed >99% living cells. Consequently, the applied experimental procedure did not affect the cells’ viability.

### 2.3. VOCs Signatures of AGS, SNU-1 and GES-1

The chemical signatures of the AGS, SNU-1 and GES-1 cells were compared to only medium using a Wilcoxon signed-rank test, and a *p*-value < 0.05 was taken as being significant. This test was used to evaluate the production and consumption of volatiles by the cells under study. Amongst all of the volatiles isolated, forty-five showed significant differences in their headspace levels compared to those above the cultivation medium only for at least one cell line of interest. More specifically, ten compounds were found to have decreased headspace concentrations, whereas the other thirty-five exhibited elevated levels in the headspace. The detection and quantification incidences of the volatiles identified, as well as their concentrations in the headspace of the cultivation flasks, are presented in Table 3. The output of the Wilcoxon signed-rank test is presented in Table 4.

The VOCs found to have decreased levels are six aldehydes (2-methylpropanal, 2-methyl-2-propenal, 2-methylbutanal, 3-methylbutanal, pentanal, and hexanal), one ketone (6-methyl-2-heptanone), two heterocyclic compounds (2-ethylfuran and 2-pentylfuran) and one ester (2-ethylhexyl ester benzoic acid). Of the volatile species with increased levels, twelve are ketones (2-butanone, 2-pentanone, 3-pentanone, 2-methyl-3-pentanone, 4-methyl-2-pentanone, 2-heptanone, cyclohexanone, 2-nonanone, 2-undecanone, 2-tridecanone, 2-pentadecanone, and 2-heptadecanone), three are esters (ethyl acetate, ethyl propanoate, and ethyl 2-methylbutyrate), six are hydrocarbons (n-pentane, 3-methylhexane, n-nonane, n-dodecane, n-tetradecane, and n-hexadecane), eight are alcohols (2-methyl-2-propanol, 1-propanol, 2-methyl-1-propanol, 2-methyl-2-butanol, 3-methyl-1-butanol, cyclohexanol, 2-ethyl-1-hexanol, and 2-methyl-1-hexadecanol), three are ethers (2-ethoxy-2-methylpropane, 2,2-dimethyloxetane, and 1,1-diethoxy ethane), and three are aromatic compounds (benzene, toluene, and styrene).

**Figure 1:**
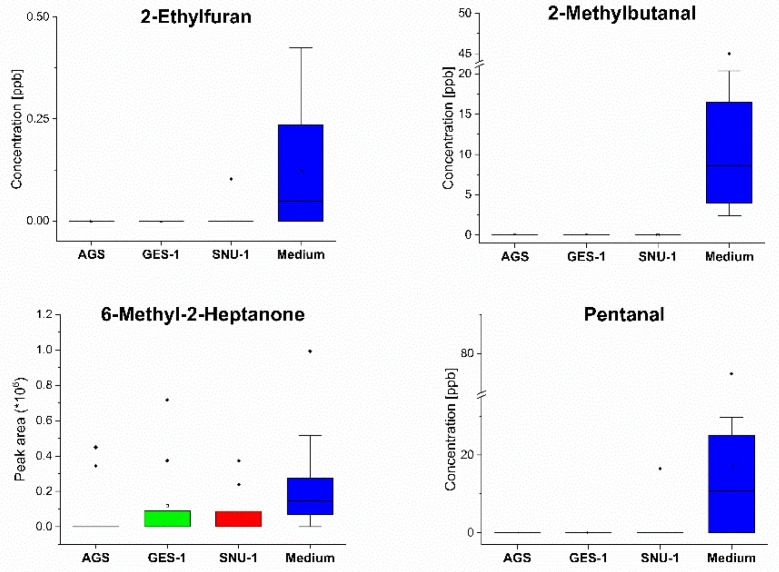
Comparison of the headspace concentrations of selected consumed VOCs over the cultures of AGS, SNU-1, GES-1 cells and medium. Red—cancer cells, green—normal cells, blue—medium.

**Figure 2:**
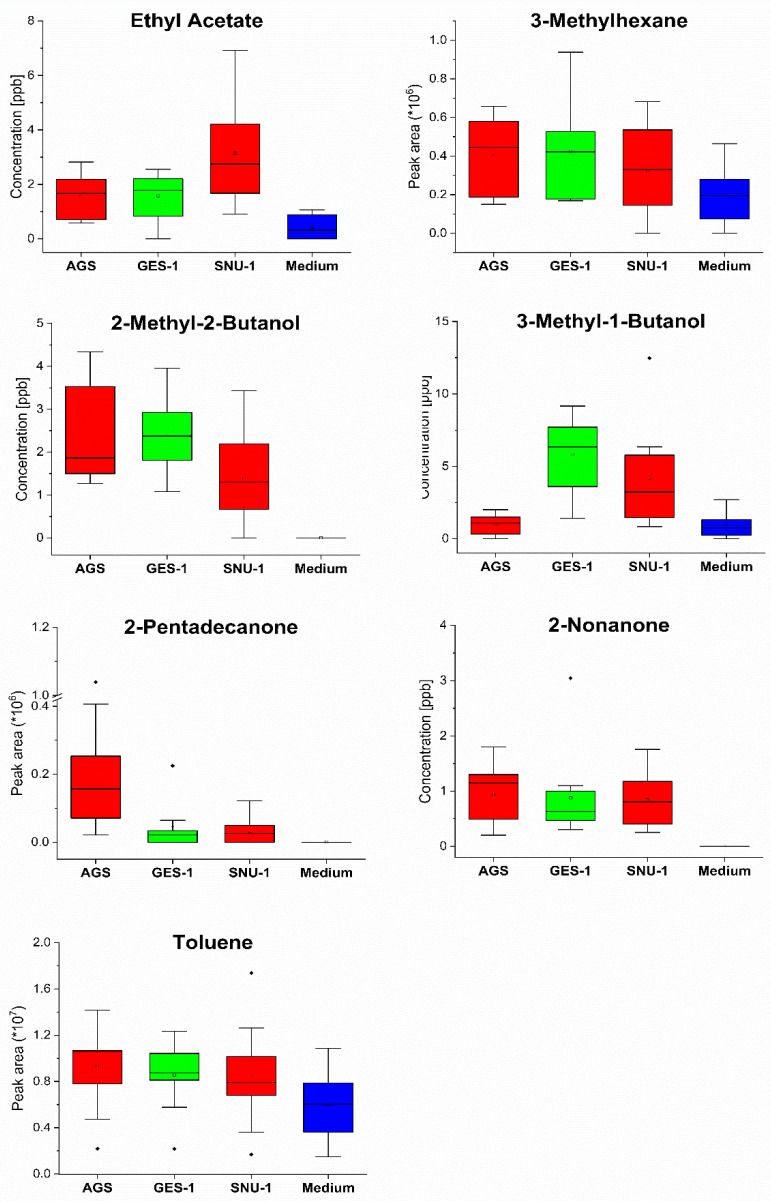
Comparison of the headspace concentrations of selected released VOCs over the cultures of AGS, SNU-1, GES-1 cells and medium. Red—cancer cells, green—normal cells, blue—medium.

An effort was made to quantify the levels of the VOCs in the various headspaces. However, this was not possible for a number of the compounds. This either was because of their unavailability as pure substances or because of problems related to the generation of reliable reference mixtures. This set of compounds embraces n-pentane (CAS:109-66-0, Rt = 2.60 min), 2,2-dimethyloxetane (CAS:6245-99-4, Rt = 7.26 min), 3-methylhexane (CAS:589-34-4 Rt = 8.66 min), benzene (CAS:71-43-2, Rt = 8.96 min), 1,1-diethoxyethane (CAS:105-57-7 Rt = 14.92 min), toluene (CAS:108-88-3, Rt = 17.60 min), 2-methyl-3-pentanone (CAS:565-69-5, Rt = 17.80 min), hexanal (CAS:66-25-1, Rt = 20.67 min), n-nonane (CAS:111-84-2, Rt = 23.8 min), styrene (CAS:100-42-5, Rt = 24.57 min), cyclohexanone (CAS:108-94-1, Rt = 25.8 min), 6-methyl-2-heptanone (CAS:928-68-7, Rt = 27.1 min), n-dodecane (CAS:112-40-3, Rt = 34.77 min), 2-undecanone (CAS:112-12-9, Rt = 38.18 min), hexadecane (CAS:544-76-3, Rt = 42.33 min), 2-ethylhexyl ester benzoic acid (CAS:5444-75-7, Rt = 44.5 min), 2-methyl-1-hexadecanol (CAS:2490-48-4, Rt = 49.98 min), 2-pentadecanone (CAS:2345-28-0, Rt = 44.26 min), and 2-heptadecanone (CAS:2922-51-2, Rt = 46.55 min). For those VOCs for which we were not able to quantify their levels directly, we assumed a linear response of the detector for the all observed concentration levels, so that their levels could be estimated from their peak areas alone. The comparison of the headspace levels of selected consumed and released VOCs is presented in Figure 1 and Figure 2, respectively. The respective comparison of the remaining volatiles is provided in Supplementary Figures S1–S8.

#### 2.3.1. Volatiles Produced and Metabolized by AGS, SNU-1 and GES-1 Cell Lines

Unsurprisingly, the dominating class of compounds being metabolized by all cells under study was found to be the aldehydes. The uptake of aldehydes characterizes numerous human cell lines, both normal and cancerous, including gastric cells [14]. In principle, two metabolic pathways are indicated as sinks of compounds from this chemical family: (i) oxidation into corresponding carboxylic acids [15,16] involving aldehyde dehydrogenases (ALDHs), and (ii) reversible reduction to alcohols by alcohol dehydrogenases (ADHs). Indeed, a number of primary alcohols, i.e., 2-methyl-1-propanol, and 3-methyl-1-butanol, resulting from the reduction of 2-methyl propanal and 3-methyl butanal, respectively, were found to be emitted by all cell cultures of interest. Moreover, 1-propanol, which is a product of propanal metabolism, was also detected in the headspace of the cell cultures and media. However, its levels exceeded the linear range of the GC-MS detector, preventing a sound comparison of the headspace levels. Unsaturated aldehydes, such as 2-methyl-2-propenal, are metabolized via conjugation with glutathione [10]. The glutathione conjugates are further metabolized by γ-glutamyl transpeptidase and cysteinyl glycinase, which is followed by acetylation. In the case of 2-methyl-2-propenal, this metabolic pathway gives 3-hydroxy-2-methylpropyl mercapturic acid (HMPMA-2).

Two heterocyclic compounds, 2-ethylfuran and 2-pentylfuran, were metabolized by the cells of interest. Although the metabolism of these species in humans is unclear, it might resemble the metabolism of 2-methylfuran. In humans, 2-methylfuran may become (i) irreversibly associated with microsomal proteins and/or DNA or (ii) oxidized by cytochrome P450 [11]. It should be stressed here, however, that the uptake of furans was also observed in other gastric cell lines, both normal and carcinogenous [17].

The metabolism of 2-ethylhexyl benzoate can be attributed to the enzymatic hydrolysis, yielding 2-ethylhexanol and benzoic acid. Indeed, the levels of 2-ethylhexanol were found to be elevated in the headspace of all of the cell cultures. Thus, 2-ethylhexyl benzoate may be considered as a source of this alcohol within this study. The second product benzoic acid can be further converted into hippuric acid [18].

Of the VOCs that are released, ketones are the dominant class with twelve representatives. Interestingly, nine of these species are methyl ketones containing (with the exception of 2 butanone) an odd number of carbons. The production of ketones can be ascribed to two potential pathways: (i) oxidation of secondary alcohols performed by ADHs and/or cytochrome P450 CYP2E1 and (ii) β-oxidation of fatty acids. Apart from the primary alcohols (mostly ethanol), ADHs are capable of oxidizing secondary, long-chain and cyclic alcohols [15,19]. Thus, 2-pentanone may stem from 2-pentanol, and 2-nonanone comes from 2-nonanol. However, none of the potential alcohol substrates for this pathway was detected in the medium or culture headspaces. Nevertheless, they could be produced by the hydroxylation of respective alkanes catalyzed by cytochrome P450 enzymes [20,21,22]. Indeed, the medium headspace was found to contain n-pentane, n-heptane, cyclohexane, n-nonane, n-undecane and n-heptadecane.

An alternative metabolic route leading to ketone formation in humans involves *β*-oxidation of the fatty acids. For example, 2-ethylhexanoic acid has been shown to be metabolized into 2-heptanone and 4-heptanone [23], and 2-pentanone is proposed to be formed via *β*-oxidation of hexanoic acid in the peroxisomal pathway [24]. Moreover, 3-pentanone is also hypothesized to be produced via the oxidation of branched-chain keto acids [13]. More specifically, it is a product of the oxidative decarboxylation of 2-methyl-3-ketovaleric acid. Perhaps other ketones observed within this study could be produced analogously?

It is worth mentioning that seven methyl ketones (namely, 2-pentanone, 2-heptanone, 2-nonanone, 2-undecanone, 2-tridecanone, 2-pentadecanone, and 2-heptadecanone) released by the cells of interest in this study were reported to be emitted by other gastric cell lines (i.e., HGC-27, CLS-145, and HSEC) [17]. Nevertheless, this is not a unique feature of gastric cancer cell lines, because methyl ketones were reported to be also produced by numerous other human cancer and normal cell lines. Examples include the following: 2-pentanone from the liver cancer cell line HepG2 [25], the lung cancer cell line A549 [26], and adipocyte cells SGBS [27]; 2-heptanone from the liver cancer line HepG2 [25]; and 2-nonanone was reported to be liberated by cell cultures of colon cancer (SW480, SW1116) [28], bladder cancer (J82, 5637) [29] and lung cancer (NCIH446) [30]. Other methyl ketones—namely, 2-undecanone, 2-tridecanone, 2-pentadecanone, and 2-heptadecanone—were identified in the cultures of bladder cancer [29], lung cancer [30,31], colon cancer [28] and prostate cancer [32]. Interestingly, 2-pentanone was found to be released in higher amounts by the gastric cancer tissues as compared to the healthy ones [33]. It is also worth noting that the AGS cells exhibited elevated emission of three heavier methyl ketones, i.e., 2-tridecanone, 2-pentadecanone, and 2-heptadecanone. Similar features characterize the metabolism of HGC-27 gastric cancer cells. Therefore, it is possible that these two lines share similar changes caused by carcinogenesis.

Three ethyl esters were found to be emitted by the cells under investigation: ethyl acetate, ethyl propanoate, and ethyl 2-methylbutyrate. However, ethyl propanoate was not produced by GES-1 cells, and ethyl 2-methylbutyrate was not released by the SNU-1 cell line. These species may stem from the esterification reaction employing ethanol (that was present in the medium headspace) and respective carboxylic acids (acetic acid, propanoic acid, and 2-methylbutanoic acid), the latter being the products of the oxidation of respective aldehydes and/or alcohols. Thus, acetic acid originates from the ethanol oxidation catalyzed by ADHs or cytochrome P450 CYP2E1 and ALDHs. However, other biochemical pathways such as the Krebs cycle or by pyruvate metabolism cannot be excluded. Analogously, propanoic acid and 2-methylbutanoic acid are presumably the products of the aforementioned oxidation of propanal and 2-methylbutanal. If so, the production of esters seems to be an indirect effect of the ALDHs activity. Ethyl esters have been reported to be liberated by several cancer cell lines. For instance, ethyl acetate is released by human breast and lung cancer cells [34,35], and ethyl propanoate in turn was detected in the cultures of breast cancer cells [35]. It is of note that all three esters were also emitted by the other gastric cancer cells investigated in our recent study (HGC-27, CLS-145, and HSEC) [17].

Regarding the alcohols, eight members of this family were found to be released. As stated before, primary alcohols most probably stem from the reduction of aldehydes performed by ADHs. For instance, 2-methylpropanal is metabolized to 2-methyl-1-propanol, and 3-methyl-1-butanol is a product of 3-methylbutanal conversion. Two potential pathways could be responsible for the production of secondary alcohols: (i) hydroxylation of respective branched alkanes [21,22] and (ii) biotransformation of ethers [12]. The hydroxylation of branched alkanes is catalyzed by cytochrome P450 isoforms, e.g., 1A2, 2B6 and 2E1, and it occurs predominantly at secondary or tertiary C-H bonds [21,36]. Thus, 2-methylpropane is converted into 2-methyl-2-propanol, and 2-methylbutane is hydroxylated into 2-methyl-2-butanol (70%) or 3-methyl-2-butanol (25%) [21]. Although 2-methylpropane is too volatile to be detected using the applied analytical method, 2-methylbutane was found in all samples. This route seems to be also responsible for the cyclohexanol formation [22,36]. An optional pathway of secondary alcohols formation employs the biotransformation of aliphatic ethers catalyzed by cytochrome P450 enzymes [12]. In this route, 2-ethoxy-2-methylpropane and 2-methoxy-2-methylpropane yield 2-methyl-2-propanol, whereas 2-methoxy-2-methylbutane is metabolized into 2-methyl-2-butanol [12]. Of the potential substrates, 2-ethoxy-2-methylpropane was found in the headspace of cells under study. The release of 2-ethyl-1-hexanol can be attributed to either one or all of the following: (i) the metabolism of di(2-ethylhexyl) phthalate (DEHP), (ii) oxidation of 2-ethyl-hexanal catalyzed by ADHs, or (iii) enzymatic hydrolysis of 2-ethylhexyl benzoate. DEHP is a plasticizer used in polyvinyl chloride products [37], which in humans is rapidly hydrolyzed to mono(2-ethylhexyl) phthalate (MEHP) and 2-ethylhexanol by cholesterol esterase (CEase), and/or carboxylesterase Ces1e. The latter is then oxidized to 2-ethylhexanoic acid and finally to 2-heptanone and 4-heptanone [23,37,38,39]. All investigated cell lines emitted 2-heptanone. The metabolic route leading to the release of 2-methyl-1-hexadecanol is unclear. However, the oxidation of 2-methylhexadecanal could produce this species.

Of all alcohols produced by the cells used in this study, two compounds (3-methyl-1-butanol and 2-ethyl-1-hexanol) were also found to be emitted by gastric cells in a recent study of ours [17]. The alcohols under scrutiny have also been reported to be liberated by several cancerous human cell lines as well as normal ones. 2-Ethyl-1-hexanol is liberated by NCI-H2087 lung cancer cells [40], while cyclohexanol is liberated by Lu7387 lung cancer cells [34], 3-methyl-1-butanol is liberated by RGP and Mm melanoma cells [41] as well as by SW1116 colon cancer cells [28]; whereas 2-methyl-2-propanol is released by Lu7466 lung cancer cells [42]. Furthermore, 1-propanol was reported to be associated with the A549, Lu7466 and Lu7387 lung cancer cells’ metabolism [34,42]. When it comes to normal cells, 3-methyl-1-butanol, 2-methyl-2-propanol, and 2-methyl-1-propanol are emitted by HBEpC and hFBÂ lung cells [26].

Three aromatic compounds, benzene, toluene and styrene, were observed to be liberated by all cells under scrutiny. The origin of these VOCs coming from the cell cultures is unclear. Nevertheless, the emission of aromatic compounds are reported in several other studies. For instance, benzene and styrene are reported to be liberated by A549 lung cancer cells [34,43], and toluene was found to be emitted by human endothelial cells (HUVEC) [44]. Moreover, benzene is reported to have been released by the gastric cancer cell line HGC-27 [17].

Six hydrocarbons were found to be liberated by the cells under study. The emission of n-pentane might mirror oxidative stress, inducing the peroxidation of unsaturated fatty acids. There is evidence that lipid peroxidation of ω3 and ω6 fatty acids leads to the production of ethane and n-pentane [45,46]. More specifically, ethane and n-pentane are generated via the β-scission of alkoxy radicals formed by the homolytic cleavage of fatty acids hydroperoxides. For example, in vitro studies have shown the production of n-pentane from linoleic and arachidonic acids [46]. The metabolic pathways leading to the formation of the remaining hydrocarbons (3-methylhexane, n-nonane, n-dodecane, n-tetradecane, and n-hexadecane) remain unclear, but it can be assumed that they are produced during lipid peroxidation processes. Interestingly, these hydrocarbons are rarely reported to be released by human cell lines [14].

The metabolic route leading to the production of the ethers, 2-ethoxy-2-methylpropane, 2,2-dimethyloxetane, and 1,1-diethoxy ethane, is unknown.

**Figure 3:**
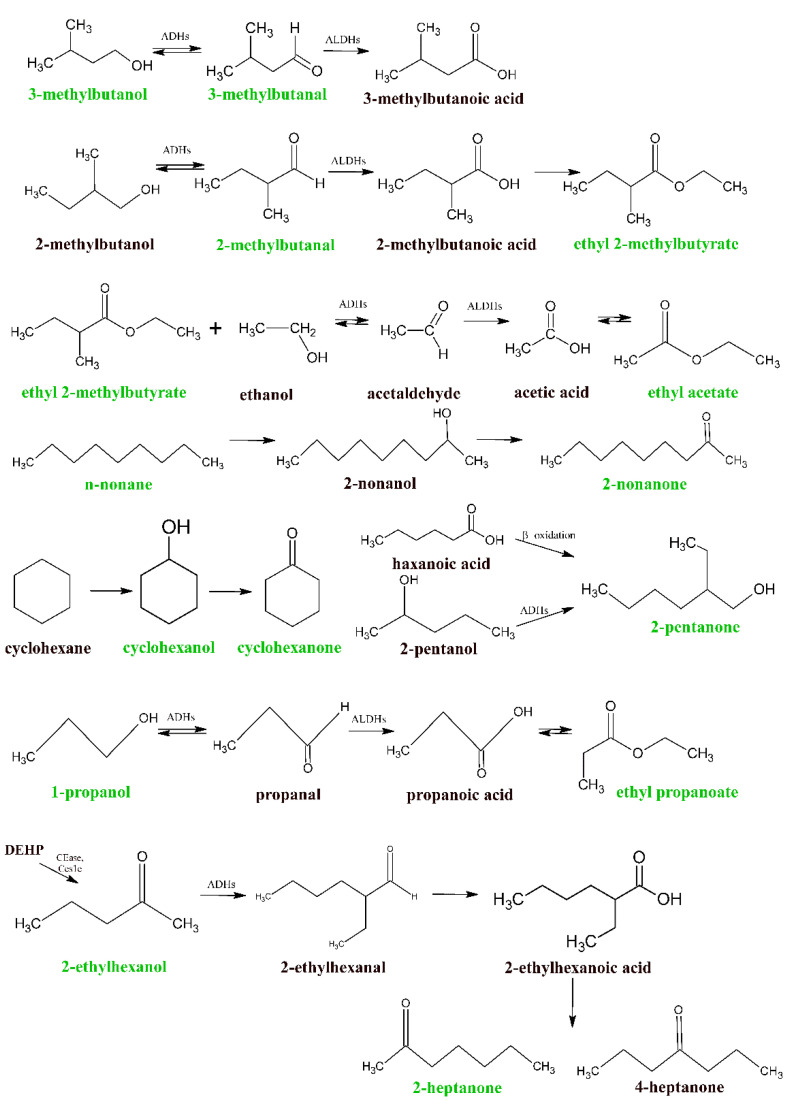
The proposed pathways leading to the consumption or production of some VOCs under study.

Some possible pathways and interrelations between the VOCs of interest are depicted in Figure 3.

#### 2.3.2. Comparison of Volatilomic Signatures of AGS, SNU-1 and GES-1 Cell Lines

To compare the production of volatiles under study, the respective signal intensities were normalized to the number of cells in particular cultures. The emission was evaluated using a Wilcoxon signed-rank test, and its outcome is presented in Table 5.

Scrutiny of Table 5 reveals some interesting information on the emission of volatiles by the cells. First, the SNU-1 cancer cells exhibited a lowered production of several alcohols in comparison to the normal ones. More specifically, this concerns 2-methyl-1-propanol, 2-methyl-2-butanol, 3-methyl-1-butanol, and cyclohexanol. Interestingly, the downregulated production of alcohols was not so pronounced in the AGS line. For this line, reduced emissions were noted only for 3-methyl-1-butanol and 2-ethyl-1-hexanol. It is of note that the analogous change in alcohols metabolism was also observed for HGC-27 and CLS-145 cell lines [17]. Thus, the downregulated production of alcohols is a common feature of gastric cancer cells metabolism. Perhaps this feature stems from the overexpression of ALDHs observed by several authors in gastric cancer cells [47,48]. Thus, aldehydes present in the medium would be preferably metabolized into carboxylic acids rather than into respective alcohols. Alternatively, this alteration could be explained by the lowered activity of ADHs. Second, the SNU-1 cells showed higher headspace concentrations of ethyl acetate and ethyl propanoate than the GES-1 and AGS lines, while AGS produced higher amounts of only one ester—ethyl acetate. The upregulated production of esters confirms the aforementioned hypothesis of the overexpression of ALDHs in the cancer lines under study. The elevated levels of acetic acid and propanoic acid would facilitate the production of esters. Furthermore, the lower production of esters by the AGS line as compared to SNU-1 agrees with the analogous differences in the alcohols production by these cells. Third, the feature of the SNU-1 volatile fingerprint is the downregulated production of ketones. The emissions of 2-butanone, 2-pentanone, 4-methyl-2-pentanone, and cyclohexanone were significantly lower from this line than from the GES-1 line. Perhaps this change is a manifestation of a lowered activity of ADHs. On the other hand, the AGS line exhibited an elevated emission of some methyl ketones. Three methyl ketones, 2-undecanone, 2-tridecanone, and 2-heptadecanone, were found to be produced predominantly by this cell line. One possible explanation of this feature may be the overexpression of ADHs in AGS, which convert primary alcohols into aldehydes and secondary alcohols into ketones. Indeed, the total ADH activity has been demonstrated to be significantly elevated in cancer tissues in general [49] and in gastric cancer tissue in particular [50]. However, alternative routes such as the upregulation of the β-oxidation of fatty acids cannot be excluded. Interestingly, a very similar overproduction of heavier methyl ketones was found for HGC-27 gastric cancer cells in our recent study [17].

When it comes to hydrocarbons, the results are ambiguous. The AGS cancer cell line showed a lowered emission of n-dodecane, whereas the SNU-1 cells were found to overproduce n-hexadecane and release lower amounts 3-methyl-hexane as compared to the GES-1 line. Since the metabolic pathways leading to the production of hydrocarbons by cells of interest are unclear, it is difficult to interpret these findings. Finally, the SNU-1 line was found to emit smaller amounts of 1,1-diethoxy-ethane than the other cells.

Studies focused on volatilomic footprints of gastric cancer cells are relatively rare. VOCs emitted by gastric cancer cells MGC-803 and GES-1 gastric mucous cells were investigated by Zhang et al., [51]. Six volatile species, namely formic acid propyl ester, 1,4-butanediol, isopropoxybutanol, nonanol, 4-butoxy-1-butanol, and 2,6,11-trimethyl dodecane, were associated with the metabolism of GES-1 cells. Three volatiles, formic acid propyl ester, 1,4-butanediol, and 2,6,11-trimethyl dodecane, were found exclusively to be produced by the GES-1 cells, whereas a further two species (butanone and 3-octanone) were detected solely in the headspace of the MGC-803 cells. The remaining three volatiles (4-isopropoxybutanol, nonanol, and 4-butoxy-1-butanol) were found to be released by both lines. However, lower emission levels came from cancer cells than from normal ones. Although these three compounds were not detected in this current study, their lowered emission by cancer cells seems to confirm additionally the downregulation of the alcohols production in gastric cancer cells.

The carcinogenesis of gastric cancer is very complex and includes not only genetic susceptibility and environmental circumstances but also a variety of acquired mutations such as chromosomal instability, microsatellite instability, somatic mutations, and epigenetic mutations. Genes affected by these factors are involved in all kind of pathways such as inflammatory response, cell growth, cell adhesion, apoptosis, DNA damage repair, and the metabolism of, e.g., foliate, polycyclic aromatic hydrocarbons, xenobiotics, or hormones [52]. As for the aforementioned cell lines HGC-27 and AGS, no microsatellite instability has been reported [53], but various genes are affected by mutations (875 mutations for HGC-27 and 1039 mutations for AGS) [54]. Both cell lines show mutations of PIK3CA, which encodes for phosphatidylinositol-4,5-bisphosphate 3-kinase and plays a critical role in the PI3K/AKT signaling pathway regulating cell growth and proliferation [55]. Another gene which is mutated in both cell lines is APC, which is a tumor suppressor that is among the five most frequently affected genes in gastric cancer [56], and it serves as a regulator of cell proliferation. There are also differences concerning mutations of characteristic genes for gastric cancer between the two cell lines. The tumor suppressor gene TP53 for instance, which regulates cell proliferation processes and maintains genomic integrity and stability [57], is mutated in about 50% of all gastric cancers [58] and therefore is the most common mutated gene in this disease. The HGC-27 cell line shows a frameshift mutation in the TP53 gene, whereas in the AGS cell line, TP53 is not affected [54]. In AGS cells, but not in the HGV-27 cell line, KRAS and CTNNB1 are both mutated. KRAS is an important mediator in the receptor tyrosine kinase pathway, which regulates cell growth and proliferation and is mutated or amplified in many human cancers, including gastric cancer [59]. CTNNB1 encodes for β-catenin, which, together with APC, plays a crucial role in the Wnt pathway regulating cell proliferation, differentiation, and migration [60]. The findings of this study indicates some similarities between AGS and HGC-27 mutations, which may go beyond the typical cancer associated mutations such as p53 or PIK3CA. Thus, more investigations are needed to relate the volatile fingerprint to tumor-specific gene mutations.

## 3. Materials and Methods

### 3.1. Chemicals and Standards

All calibration mixtures were made using high-purity liquid substances and a protocol outlined in detail elsewhere [25,61]. Therefore, only a brief description of the procedure is required here. Reference chemicals with stated purities of 95–99.9% were purchased from Merck (Darmstadt, Germany) and Fluka (Buchs, Switzerland). Calibration mixtures were prepared by injecting and evaporating several microliters of the liquid compound into evacuated and heated 1 L glass bulbs (Supelco, Bellefonte, ON, Canada). Different calibration concentrations were obtained by transferring appropriate volumes from the bulb mixtures into Tedlar bags (SKC Inc., Eighty Four, PA, USA), which were pre-filled with purified and humidified air (RH 100% at 34 °C). Ultimately, gas mixtures with VOC volume fractions ranging from 0.07 to 40 parts per billion by volume (ppbv) were used for calibration. The calibration curves relied on the use of seven distinct and independent concentration levels.

### 3.2. Cells’ Cultivation

Within this study, two gastric carcinoma cell lines, AGS and SNU-1, as well as the non-tumorigenic cell line GES-1, were used. The AGS cell line was derived from stomach tumor fragments of a 54-year-old female patient with no prior therapy. SNU-1 was established from a 44-year-old male gastric cancer patient with poorly differentiated primary carcinoma of the stomach prior to cytotoxic therapy. GES-1 is an immortalized and non-tumorigenic human gastric epithelial cell line derived from fetal stomach epithelium and served as the control for this study.

AGS and GES-1 cell lines are both adherent cell lines, whereas SNU-1 grows in multicellular aggregates in suspension. All cells were checked for mycoplasma contaminations using a MycoSPY R-PCR-Kit (Biontex, Munich, Germany) together with a LightCycler^®^ 480 Real-Time PCR System (Roche Diagnostics, Vienna, Austria).

Cell lines were cultivated at 37 °C under a humidified atmosphere with 5% CO_2_ in a medium containing 45% DMEM/F12 medium (Thermo Fisher Scientific, Waltham, MA, USA), 45% Ham’s F-12K (Kaighn’s) medium (Thermo Fisher Scientific), and 10% FCS (Thermo Fisher Scientific, #10270106). Doubling times under the cell culture conditions described above were about 24 h for all three cell lines. Three days prior to measurements, 3.5 × 10^6^ cells were seeded in a 100 mL cell culture medium contained in glass flasks (Ruprechter, Breitenbach, Austria), which have a volume of 21 × 5.5 × 11.5 cm^3^ (1 L nominal volume, bottom area of ≈240 cm^2^). Each experiment consisted of all three cell lines as well as a medium control. In total, 10 (A–J) different experiments at different time points were performed.

### 3.3. HS-NTE Sampling Protocol

In this study, we used headspace needle trap extraction (HS-NTE) as the pre-concentration method of volatiles contained in the headspace above the cultivating medium only and above cells in the cultivating medium. Since the applied HS-NTE extraction protocol has been described elsewhere [25,27], only a brief summary will be presented here. Two-bed 23-gauge Silcosteel-treated stainless steel needle trap devices (NTDs) (2 cm of Carbopack X and 1 cm of Carboxen 1000, both 60/80 mesh, PAS Technology, Magdala, Germany) were used for this purpose. Prior to the extraction, NTDs were pre-conditioned at 290 °C under a flow of high-purity nitrogen (99.9999%) for 10 min. The NTE was performed by introducing an NTD through a septum into the cultivation bottle and collecting 80 mL of the headspace gas at a constant flow rate of 3 mL min^−1^. After extraction, a given needle was inserted into the inlet of the gas chromatograph, where trapped volatiles were thermally desorbed at 290 °C in the splitless mode. For each replicate, one blank sample containing nitrogen was analyzed using the analogous protocol to identify possible contaminants from sources other than from cells or the medium. If applicable, the resulting concentration levels were subtracted from the corresponding values in the associated headspace samples.

### 3.4. GC-MS Analysis

GC-MS analysis relied on an Agilent 8890/7079B GC-MS (Agilent, Santa Clara, CA, USA). The GC injector was equipped with an inert SPME liner (inner diameter 0.75 mm, Supelco, Bellefonte, ON, Canada) and operated in the splitless mode (0.75 min) followed by split mode with a ratio of 1:50. Extracted compounds were separated using a Rxi-624Sil MS column (30 m × 0.32 mm, layer thickness 1.8 μm, Restek, Bellefonte, PA, USA) operated in constant helium flow at 1.4 mL min-1. The column temperature program was as follows: 37 °C for 12 min, followed by 5 °C min-1 up to 150 °C, then 10 °C min^−1^ up to 290 °C, and finally remaining at 290 °C for 8 min. The untargeted VOC analysis relied on the mass spectrometer working in a SCAN mode with the associated *m*/*z* ranging from 20 up to 250. The peak integration was based on extracted *m*/*z* ratio chromatograms, and such an approach allowed for a separation of the majority of peaks of interest from their neighbors. The quadrupole rods, ion source, and transfer line were kept at 150 °C, 230 °C, and 280 °C, respectively.

VOC identification was performed using a two-step process. First, the spectrum of a peak was checked against the NIST mass spectral library database. Next, the NIST identification was confirmed by comparing the retention times of peaks of interest with retention times obtained for reference standards prepared as outlined above.

## 4. Conclusions

The main goal of this study was to determine the cancer-induced changes in the volatilomic signatures of human AGS and SNU-1 gastric cancer cell lines. Amongst all of the volatiles isolated, forty-five showed significant differences in their headspace levels compared to the volatiles above the headspace of the cultivating medium only. Of the VOCs identified, ten were found to be metabolized, and thirty-five were found to be produced. The SNU-1 cell line was found to have significantly altered the VOCs’ metabolism in comparison to normal GES-1 cells. This was manifested by the decreased production of alcohols and ketones. Only esters exhibited the opposite alteration in the SNU-1 line. While the downregulated production of alcohols could result from the overexpression of ALDHs, the reduced emission of ketones may stem from the lowered activity of ADHs. The AGS cells do not show such pronounced alterations. For example, the lowered emission of alcohols was noted only for two species, 3-methyl-1-butanol and 2-ethyl-1-hexanol. However, one distinctive feature of their metabolism is the increased production of three methyl ketones containing an odd number of carbons, namely 2-tridecanone, 2-pentadecanone, and 2-heptadecanone.

Although the underlying metabolic routes to the production of VOCs of interest are not elucidated in sufficient depth, the results of this study provide evidence that the cancer state modifies the volatilome of human cells, and that the cancer-related changes are detectable using chemical analysis of respective chemical volatile signatures. The applicability of VOCs found in this study as biomarkers for gastric cancer diagnosis remains to be clarified in future studies.

## Figures and Tables

**Table 1:** Retention times (R_t_) (min), quantifier ions, LODs (ppb), RSDs (%), coefficients of variation (R^2^), Anderson-Darling normality test *p*-values (A–D) and linear ranges (ppb) for compounds of interest. Compounds are ordered with respect to increasing retention time.

VOC	CAS	R_t_ (min)	Quantifier Ion	LOD (ppb)	RSD (%)	R^2^	A–D *p*-Value	Linear Range (ppb)
2-Propanol, 2-methyl-	75-65-0	4.17	59	0.04	9.0	0.995	0.25	0.13–20
Propanal, 2-methyl-	78-84-2	4.67	72	0.04	9.0	0.981	0.12	0.13–16
2-Propenal, 2-methyl-	78-85-3	5.01	70	0.06	10	0.990	0.27	0.18–30
1-Propanol	71-23-8	5.52	59	0.09	10	0.993	0.11	0.31–37
Propane, 2-ethoxy-2-methyl-	637-92-3	6.13	59	0.05	13	0.990	0.27	0.16–19
2-Butanone	78-93-3	6.50	72	0.22	10	0.989	0.27	0.73–60
Ethyl acetate	141-78-6	6.71	43	0.04	7.0	0.983	0.14	0.12–13
1-Propanol, 2-methyl-	78-83-1	9.40	43	0.13	13	0.997	0.14	0.46–20
2-Butanol, 2-methyl-	75-85-4	9.80	59	0.09	13	0.994	0.27	0.3–19
Butanal, 3-methyl-	590-86-3	9.97	58	0.06	9.0	0.981	0.27	0.17–50
Butanal, 2-methyl-	96-17-3	10.57	57	0.02	9.0	0.990	0.28	0.12–30
Furan, 2-ethyl-	3208-16-0	12.10	81	0.04	10	0.990	0.22	0.12–6
2-Pentanone	107-87-9	13.30	43	0.08	12	0.995	0.13	0.26–28
n-Pentanal	110-62-3	13.80	58	2	10	0.996	0.55	6–30
3-Pentanone	96-22-0	14.03	57	0.03	13	0.990	0.15	0.09–20
Ethyl propanoate	105-37-3	14.20	57	0.04	11	0.979	0.34	0.12–13
2-Pentanone, 4-methyl-	108-10-1	17.30	43	0.07	12	0.987	0.07	0.2–17
1-Butanol, 3-methyl-	123-51-3	18.20	55	0.07	12	0.994	0.16	0.23–17
Ethyl 2-methylbutyrate	7452-79-1	22.54	102	0.03	11	0.982	0.36	0.09–21
2-Heptanone	105-42-0	25.17	58	0.04	13	0.988	0.11	0.12–11
Cyclohexanol	108-93-0	25.40	57	0.02	7.0	0.990	0.39	0.07–24
2-Pentylfuran	3777-69-3	28.19	81	0.02	7.0	0.989	0.43	0.08–3
1-Hexanol, 2-ethyl-	104-76-7	30.73	57	0.04	7.0	0.998	0.23	0.12–60
2-Nonanone	821-55-6	32.72	58	0.03	14	0.994	0.19	0.08–9.5
n-Tetradecane	629-59-4	39.20	57	0.06	14	0.980	0.64	0.2–19
2-Tridecanone	593-08-8	41.60	58	0.13	15	0.988	0.16	0.45–9.5

**Table 2:** Total number of cells (×10^6^) in the cultivation flasks at the time of the measurement.

Line	Total Number of Cells (×10^6^)									
	A	B	C	D	E	F	G	H	I	J
AGS	13.5	47.5	40.4	33.3	43.1	30.1	19.8	39.1	23.8	48.7
SNU-1	14.7	27.2	27.7	49.5	43.0	42.0	36.5	66.5	27.3	39.2
GES-1	20.8	37.4	19.3	16.5	38.1	22.5	30.6	48.7	35.5	45.8

**Table 3:** Detection (n_d_) and quantification (n_q_) incidences, concentration ranges, and medians (for calibrated compounds) of VOCs in the headspace of media and cell cultures. Compounds are ordered with respect to increasing retention time. Compounds in italics were not quantified for reasons mentioned in the text.

	VOC	CAS	AGS		SNU-1		GES-1		Medium	
			Incidence n_d_ (n_q_)	Range (Median) (ppb)	Incidence n_d_ (n_q_)	Range (Median) (ppb)	Incidence n_d_ (n_q_)	Range (Median) (ppb)	Incidence n_d_ (n_q_)	Range (Median) (ppb)
Uptake	Propanal, 2-methyl	78-84-2	0	-	1/1	0.21	1/1	0.4	8/8	0.32–18 (8.7)
	2-Propenal, 2-methyl-	78-85-3	0	-	0	-	0	-	8/8	0.4–30 (2.4)
	Butanal, 3-methyl-	590-86-3	0	-	8	2.2–10 (2.3)	1	2.3	10/10	14–110 (70)
	Butanal, 2-methyl-	96-17-3	0	-	0	-	0	-	10/10	2.4–45 (8.6)
	Furan, 2-ethyl-	3208-16-0	0	-	1/0	-	0	-	5/5	0.12–0.43 (0.24)
	Pentanal	110-62-3	0	-	1/1	6.5	0	-	6/6	6.2–75 (10.9)
	Hexanal	66-25-1	0	-	0	-	0	-	6	-
	2-Heptanone, 6-methyl-	928-68-7	2	-	4	-	3	-	9	-
	2-Pentylfuran	3777-69-3	0	-	3	0.37–1.1 (0.51)	2/2	0.3–1.3	7	0.43–1.4 (0.74)
	Benzoic acid, 2-ethylhexyl ester	5444-75-7	4	-	0	-	1	-	7	-
Release	n-Pentane	109-66-0	10	-	10	-	10	-	10	-
	2-Propanol, 2-methyl-	75-65-0	10/10	1.5–25 (3.21)	10/10	0.3–15 (2.4)	10/10	1.4–24 (2.26)	9/9	0.36–9.0 (0.76)
	1-Propanol	71-23-8	7/7	3–258 (7.0)	8/8	3.0–151 (6.0)	8/8	2.5–228 (4.7)	6/6	1.6–167 (4.5)
	Propane, 2-ethoxy-2-methyl-	637-92-3	9/9	2.4–38 (11)	10/10	2.5–40 (5.8)	9/9	2.2–43 (16.7)	7/7	2.5–22 (4.2)
	2-Butanone	78-93-3	10	69–193 (120)	10	37–170 (99)	10	68–194 (127)	10	40–158 (82)
	Ethyl acetate	141-78-6	10/10	0.6–2.8 (1.7)	10/10	0.9–6.9 (2.7)	9/9	0.75–2.6 (1.9)	6/6	0.3–0.8 (1.1)
	Oxetane, 2,2-dimethyl-	6245-99-4	7	-	3	-	7	-	0	-
	Hexane, 3-methyl-	589-34-4	10	-	9	-	10	-	8	-
	Benzene	71-43-2	10	-	10	-	10	-	9	-
	1-Propanol, 2-methyl-	78-83-1	10/10	4.7–15 (6.9)	9/9	1.4–12 (4.9)	10/10	3.8–14 (7.14)	9/9	1.4–4.3 (2.6)
	2-Butanol, 2-methyl-	75-85-4	10/10	1.3–4.3 (1.9)	9/9	0.34–3.4 (1.3)	10/10	1.1–4.0 (2.4)	0	-
	2-Pentanone	107-87-9	10/10	1.9–12 (4.2)	10/10	0.9–9.0 (3.3)	10/10	2–12 (3.6)	10/10	0.5–5.8 (1.8)
	Ethyl propanoate	105-37-3	8/6	0.3–0.5 (0.36)	10/9	0.18–1.9 (1.0)	0	-	2/0	-
	3-Pentanone	96-22-0	9/9	0.4–0.8 (0.6)	9/9	0.25–0.74 (0.5)	10/10	0.3–0.7 (0.5)	8/8	0.2–0.6 (0.47)
	Toluene	108-88-3	10	-	10	-	10	-	10	-
	3-Pentanone, 2-methyl-	565-69-5	6	-	0	-	4	-	0	-
	1-Butanol, 3-methyl-	123-51-3	9/9	0.22–2 (1.2)	10/10	0.8–12 (3.2)	10/10	1.4–9 (6.34)	8/8	0.25–2.7 (1.0)
	Ethane, 1,1-diethoxy-	105-57-7	9	-	8	-	8	-	7	-
	2-Pentanone, 4-methyl-	108-10-1	9/9	0.3–6.4 (0.92)	9/8	0.2–9.4 (0.85)	10/10	0.2–7.8 (0.68)	9/8	0.2–5.3 (0.75)
	Ethyl 2-methylbutyrate	7452-79-1	8/6	0.15–0.68 (0.33)	5/2	0.04-0.4	8/6	0.1–0.3 (0.21)	0	-
	Styrene	100-42-5	10	-	10	-	10	-	10	-
	2-Heptanone	105-42-0	10/8	0.48–0.66 (0.57)	10/8	0.16–1.1 (0.56)	10/8	0.3–1.1 (0.5)	7/6	0.14–0.45 (0.2)
	n-Nonane	111-84-2	10	-	10	-	10	-	9	-
	Cyclohexanol	108-93-0	10/10	11–37 (20)	10/10	9–32 (16)	10/10	11–36 (18)	10/10	8–34 (16)
	Cyclohexanone	108-94-1	10	-	10	-	10	-	9	-
	1-Hexanol, 2-ethyl-	104-76-7	10/10	6–67 (39)	10/10	11–102 (44)	10/10	28–122 (47)	10/10	7–32 (15.7)
	2-Nonanone	821-55-6	10/10	0.2–1.8 (1.2)	10/10	0.25–1.8 (0.8)	10/10	0.3–3 (0.6)	0	-
	n-Dodecane	112-40-3	10	-	10	-	10	-	10	-
	2-Undecanone	112-12-9	5	-	0	-	6	-	0	-
	n-Tetradecane	629-59-4	10/10	9–49 (20.5)	10/10	13–46 (27.6)	10/10	5–39 (17.7)	10/10	5.5–13 (13)
	2-Tridecanone	593-08-8	7/7	3.2–8.6 (3.7)	3/3	2.1–5.5 (2.8)	3/3	2.0–11 (2.7)	0	-
	n-Hexadecane	544-76-3	4	-	8	-	4	-	3	-
	2-Pentadecanone	2345-28-0	10	-	6	-	7	-	0	-
	2-Heptadecanone	2922-51-2	8	-	0	-	0	-	0	-
	1-Hexadecanol, 2-methyl-	2490-48-4	8	-	2	-	2	-	1	-

**Table 4:** Consumption and emission of VOCs by AGS, SNU-1, and GES-1 cells related to the medium only and tentative metabolic pathways of their production. *p*-values refer to the Wilcoxon signed-rank test. Compounds in italics were not quantified for reasons mentioned in the text. n.s.: not significant.

	VOC	CAS	AGS	SNU-1	GES-1	Tentative Metabolic Pathways	
			*p*-Value	*p*-Value	*p*-Value	Tentative Product(s)	Enzyme/Other
Uptake	Propanal, 2-methyl	78-84-2	7.1 × 10^−3^	6.4 × 10^−3^	7.1 × 10^−3^	I. 2-Methylpropanol, II. 2-Methyl propanic acid	I. ADHs II. ALDHs
	2-Propenal, 2-methyl-	78-85-3	7.1 × 10^−3^	7.1 × 10^−3^	7.1 × 10^−3^	3-Hydroxy-2-methylpropyl mercapturic acid [10]	γ-glutamyl transpeptidase, cysteinyl lycinase, N-acetyl transferase, reductasesa
	Butanal, 3-methyl-	590-86-3	9.8 × 10^−4^	9.8 × 10^−4^	9.8 × 10^−4^	I.3-Methylbutanol, II. 3-Methyl butanoic acid	I. ADHs II. ALDHs
	Butanal, 2-methyl-	96-17-3	9.8 × 10^−4^	9.8 × 10^−4^	9.8 × 10^−4^	I. 2-Methylbutanol, II. 2-Methyl butanoic acid	I. ADHs II. ALDHs
	Furan, 2-ethyl-	3208-16-0	0.03	0.05	0.03	association with microsomal proteins and/or DNA [11]	Cytochrome P450 (2E1)
	Pentanal	110-62-3	0.02	0.04	0.02	I. 1-Pentanol II. Pentanoic acid	I. ADHs II. ALDHs
	Hexanal	66-25-1	0.03	0.03	0.03	I. 1-Hexanol, II. Hexanoic acid	I. ADHs II. ALDHs
	2-Heptanone, 6-methyl-	928-68-7	0.02	0.05	n.s.	6-Methyl-2-heptanol	ADHs
	2-Pentylfuran	3777-69-3	0.01	n.s.	0.05	association with microsomal proteins and/or DNA [11]	Cytochrome P450 (2E1)
	Benzoic acid, 2-ethylhexyl ester	5444-75-7	0.02	0.01	0.01	Benzoic acid and 2-ethyl-1-hexanol	Carboxylesterases
	n-Pentane	109-66-0	0.01	0.01	0.01	lipids	Oxidative stress, in vivo lipid peroxidation
	2-Propanol, 2-methyl-	75-65-0	9.8 × 10^−4^	0.01	0.03	I. 2-Methoxy-2-methylpropane/ 2-Ethoxy-2-methyl-propane [12] II. 2 Methylpropane	I. monoxygenase e.g., cytochrome P-450 2A6 II. hydroxylation catalyzed by cytochrome p450 isoforms (1A2, 2B6, and 2E1)
	1-Propanol	71-23-8	0.01	n.s.	0.01	Propanal	ADHs
	Propane, 2-ethoxy-2-methyl-	637-92-3	0.03	n.s.	0.01	unknown	unknown
	2-Butanone	78-93-3	9.8 × 10^−4^	n.s.	9.8 × 10^−4^	I. 2-Butanol II. fatty acids	I. ADHs and/or cytochrome p450 CYP2E1 II. β-oxidation
	Ethyl acetate	141-78-6	9.8 × 10^−4^	9.8 × 10^−4^	4.6 × 10^−3^	Ethanol + acetic acid	esterification
	Oxetane, 2,2-dimethyl-	6245-99-4	0.01	-	0.01	unknown	unknown
	Hexane, 3-methyl-	589-34-4	0.01	n.s.	1.9 × 10^−3^		
	Benzene	71-43-2	1.9 × 10^−3^	9.8 × 10^−4^	2.9 × 10^−3^	unknown	unknown
	1-Propanol, 2-methyl-	78-83-1	9.8 × 10^−4^	6.4 × 10^−3^	9.8 × 10^−4^	I. 2-Methyl-propanal	ADHs
	2-Butanol, 2-methyl-	75-85-4	9.8 × 10^−4^	4.6 × 10^−3^	9.8 × 10^−4^	I. Tert-amyl methyl ether [12] II. 2-Methylbutane	I. monoxygenase e.g., cytochrome P-450 II. hydroxylation catalyzed by cytochrome p450 isoforms (1A2, 2B6, and 2E1)
	2-Pentanone	107-87-9	9.8 × 10^−4^	4.9 × 10^−3^	9.8 × 10^−4^	I. 2-Pentanol II. fatty acids: hexanoic acid	I, ADHs and/or cytochrome p450 CYP2E1 II. β-oxidation
	Ethyl propanoate	105-37-3	7.8 × 10^−4^	2.0 × 10^−3^	n.s.	Ethanol +propanoic acid	esterification
	3-Pentanone	96-22-0	6.4 × 10^−3^	0.03	2.9 × 10^−3^	2-Methyl- 3-ketovaleric acid [13]	propionyl-CoA/methylmalonyl-CoA
	Toluene	108-88-3	4.8 × 10^−3^	4.8 × 10^−3^	6.8 × 10^−3^	unknown	unknown
	3-Pentanone, 2-methyl-	565-69-5	0.02	n.s.	n.s.	2-Methyl-3-pentanol	ADHs and/or cytochrome p450 CYP2E1
	1-Butanol, 3-methyl-	123-51-3	n.s.	1.9 × 10^−3^	1.9 × 10^−3^	3-Methylbutanal	ADHs
	Ethane, 1,1-diethoxy-	105-57-7	4.6 × 10^−3^	n.s.	0.02	unknown	unknown
	2-Pentanone, 4-methyl-	108-10-1	0.03	n.s.	0.02	4-Methyl2-pentanol	ADHs and/or cytochrome p450 CYP2E1
	Ethyl 2-methylbutyrate	7452-79-1	7.8 × 10^−3^	n.s.	7.8 × 10^−3^	Ethanol + 2-methylbutanoic acid	esterification
	Styrene	100-42-5	0.01	4.9 × 10^−3^	9.8 × 10^−4^	unknown	
	2-Heptanone	105-42-0	1.9 × 10^−3^	1.9 × 10^−3^	1.9 × 10^−3^	I. 2-Heptanol II. fatty acids: 2-ethylhexanoic acid	I. ADHs and/or cytochrome p450 CYP2E1 II. β-oxidation
	n-Nonane	111-84-2	0.03	0.04	0.02	unknown	
	Cyclohexanol	108-93-0	0.02	n.s.	0.03	Cyclohexane (medium)	Hydroxylation by cytochrome P-450
	Cyclohexanone	108-94-1	0.03	n.s.	0.03	Cyclohexanol and cyclohexane (medium)	ADHs
	1-Hexanol, 2-ethyl-	104-76-7	2.9 × 10^−3^	9.8 × 10^−4^	1.9 × 10^−3^	I. Di(2-ethylhexyl)phtalate II. 2-Ethyl-hexanal III. 2-Ethylhexyl ester benzoic acid	I. CEase, Ces1e II. ADHs III. cholesterol esterase (CEase), and/or carboxylesterase Ces1e
	2-Nonanone	821-55-6	1.9 × 10^−3^	1.9 × 10^−3^	1.9 × 10^−3^	I. 2-Nonanol and n-nonane (medium) II. fatty acids	I. ADHs and/or cytochrome p450 CYP2E1 II. β-oxidation
	n-Dodecane	112-40-3	4.8 × 10^−3^	n.s.	n.s.	unknown	
	2-Undecanone	112-12-9	0.03	0.02	n.s.	I. 2-Undecanol II. fatty acids	I. ADHs and/or cytochrome p450 CYP2E1 II. β-oxidation
	n-Tetradecane	629-59-4	0.01	9.8 × 10^−4^	n.s.	unknown	
	2-Tridecanone	593-08-8	0.01	n.s.	n.s.	I. 2-Tridecanol II. fatty acids	I. ADHs and/or cytochrome p450 CYP2E1 II. β-oxidation
	n-Hexadecane	544-76-3	n.s.	7.1 × 10^−3^	n.s.	unknown	
	2-Pentadecanone	2345-28-0	9.8 × 10^−4^	0.02	0.01	I. 2-Pentadecanol II. fatty acids	I. ADHs and/or cytochrome p450 CYP2E1 II. β-oxidation
	2-Heptadecanone	2922-51-2	7.1 × 10^−3^	n.s.	n.s.	I. 2-Heptadecanol II. fatty acids	I. ADHs and/or cytochrome p450 CYP2E1 II. β-oxidation
	1-Hexadecanol, 2-methyl-	2490-48-4	0.04	n.s.	n.s.	unknown	

**Table 5:** Comparison of the emission of VOCs by the cells under study. *p*-values refer to the Wilcoxon signed-rank test. Compounds in italics were not quantified for reasons mentioned in the text. n.s.: not significant. ↑ upregulated, ↓ downregulated.

	VOC	CAS	AGS vs. GES-1 *p*-Value	SNU-1 vs. GES-1 *p*-Value	AGS vs. SNU-1 *p*-Value
Release	n-Pentane	109-66-0	n.s.	n.s.	n.s.
	2-Propanol, 2-methyl-	75-65-0	n.s.	n.s.	↑9.8 × 10^−4^
	1-Propanol	71-23-8	n.s.	n.s.	n.s.
	Propane, 2-ethoxy-2-methyl-	637-92-3	n.s.	n.s.	n.s.
	2-Butanone	78-93-3	n.s.	↓9.8 × 10^−4^	↑9.8 × 10^−4^
	Ethyl acetate	141-78-6	n.s.	↑9.8 × 10^−4^	↓9.8 × 10^−4^
	Oxetane, 2,2-dimethyl-	6245-99-4	n.s.	n.s.	n.s.
	Hexane, 3-methyl-	589-34-4	n.s.	↓2.9 × 10^−3^	↑0.04
	Benzene	71-43-2	n.s.		↑4.8 × 10^−3^
	1-Propanol, 2-methyl-	78-83-1	n.s.	↓1.9 × 10^−3^	↑4.8 × 10^−3^
	2-Butanol, 2-methyl-	75-85-4	n.s.	↓ 9.8 × 10^−4^	↑1.9 × 10^−3^
	2-Pentanone	107-87-9	n.s.	↓6.8 × 10^−3^	↑0.03
	Ethyl propanoate	105-37-3	↑3.9 × 10^−3^	↑9.8 × 10^−4^	↓6.8 × 10^−3^
	3-Pentanone	96-22-0	↑0.04	n.s.	↑0.04
	Toluene	108-88-3	n.s.	n.s.	n.s.
	3-Pentanone, 2-methyl-	565-69-5	n.s.	n.s.	↑0.02
	1-Butanol, 3-methyl-	123-51-3	↓9.8 × 10^−4^	↓0.03	↓4.8 × 10^−3^
	Ethane, 1,1-diethoxy-	105-57-7	n.s.	↓7.8 × 10^−3^	↑1.9 × 10^−3^
	2-Pentanone, 4-methyl-	108-10-1	↓0.02	↓0.02	n.s.
	Ethyl 2-methylbutyrate	7452-79-1	n.s.	n.s.	n.s.
	Styrene	100-42-5	n.s.	n.s.	n.s.
	2-Heptanone	105-42-0	n.s.	n.s.	n.s.
	n-Nonane	111-84-2	n.s.	n.s.	n.s.
	Cyclohexanol	108-93-0	n.s.	↓9.8 × 10^−4^	↑2.9 × 10^−3^
	Cyclohexanone	108-94-1	n.s.	↓9.8 × 10^−4^	↑9.8 × 10^−4^
	1-Hexanol, 2-ethyl-	104-76-7	↓9.7 × 10^−3^	n.s.	n.s.
	2-Nonanone	821-55-6	n.s.	n.s.	n.s.
	n-Dodecane	112-40-3	↓6.8 × 10^−3^	n.s.	↓0.01
	2-Undecanone	112-12-9	n.s.	n.s.	n.s.
	n-Tetradecane	629-59-4	n.s.	n.s.	n.s.
	2-Tridecanone	593-08-8	↑0.05	n.s.	↑0.02
	n-Hexadecane	544-76-3	n.s.	↑7.8 × 10^−3^	↓2.7 × 10^−3^
	2-Pentadecanone	2345-28-0	↑9.8 × 10^−4^	n.s.	↑9.8 × 10^−4^
	2-Heptadecanone	2922-51-2	↑3.9 × 10^−3^	n.s.	↑3.9 × 10^−3^
	1-Hexadecanol, 2-methyl-	2490-48-4	n.s.	n.s.	n.s.

## Data Availability

The data presented in this study are available on request from the corresponding author.

## Supplementary Materials

The following supporting information can be downloaded at: https://www.mdpi.com/article/10.3390/molecules27134012/s1, Figure S1: Comparison of the headspace concentrations of 1,1 diethoxyethane, 1-propanol, 2,2 dimethoxyethane, and 2-butanone over the cultures of AGS, SNU-1, GES-1 cells and medium; Figure S2: Comparison of the headspace concentrations of 2-ethoxy-2-methylpropane, 2-ethyl-1-hexanol, 2-heptadecanone, and 2-heptanone over the cultures of AGS, SNU-1, GES-1 cells and medium; Figure S3: Comparison of the headspace concentrations of 2-methyl-1-hexadecanol, 2-methyl-2-propenal, 2-methyl-1-propanol, and 2-methyl-2-propanol over the cultures of AGS, SNU-1, GES-1 cells, and medium; Figure S4: Comparison of the headspace concentrations of 2-methyl-3-pentanone, 2-methylpropanal, 2-pentanone, and 2-pentylfuran over the cultures of AGS, SNU-1, GES-1 cells, and medium; Figure S5: Comparison of the headspace concentrations of 2-tridecanone, 2-undecanone, 3-methylbutanal, and 3-pentanone over the cultures of AGS, SNU-1, GES-1 cells, and medium; Figure S6: Comparison of the headspace concentrations of 4-methyl-2-pentanone, benzene, cyclohexanol, and cyclohexanone over the cultures of AGS, SNU-1, GES-1 cells, and medium; Figure S7: Comparison of the headspace concentrations of n-dodecane, ethyl 2-methylbutyrate, ethyl propanoate, and hexanal over the cultures of AGS, SNU-1, GES-1 cells, and medium; Figure S8: Comparison of the headspace concentrations of n-hexadecane, n-nonane, styrene, and n-tetradecane over the cultures of AGS, SNU-1, GES-1 cells, and medium; Figure S9: Exemplary chromatogram from HS-NTE-GCMS analysis of an AGS cell culture head-space; Figure S10: Exemplary chromatogram from HS-NTE-GCMS analysis of a SNU-1 cell culture head-space; Figure S11: Exemplary chromatogram from HS-NTE-GCMS analysis of a GES-1 cell culture head-space; Figure S12: Exemplary chromatogram from HS-NTE-GCMS analysis of a medium head-space.
