# Supplementary material for: Volatilomic Signatures of AGS and SNU-1 Gastric Cancer Cell Lines
Source: Molecules. 2022 Jun 22;27(13):4012. doi: 10.3390/molecules27134012 (PMC9268292; doi:10.3390/molecules27134012)
Supplement: Supplementary file 1 [file molecules-27-04012-s001.zip › molecules-1753292-supplementary.pdf]

Supplementary Materials

for the article:

Volatilomic signatures of AGS and SNU-1 gastric cancer cell lines

Daria Ślefarska-Wolak, Christine Heinzle, Andreas Leiherer, Clemens Ager, Axel Muendlein, Linda Mezmale[6,8], Marcis Leja, Alejandro H. Corvalan, Heinz Drexel, Agnieszka Królicka, Gidi Shani, Chris A. Mayhew, Hossam Haick, Pawel Mochalski

Submitted to:

Molecules

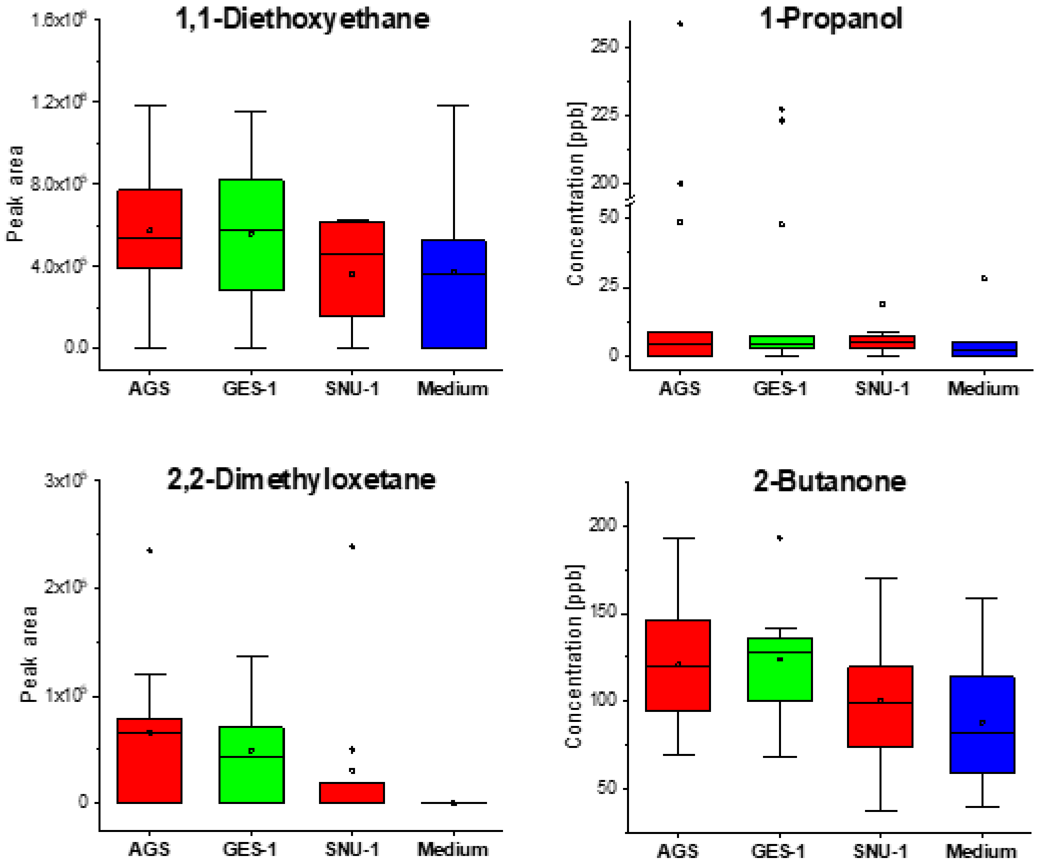

Figure S1. Comparison of the headspace concentrations of 1,1 diethoxyethane, 1-propanol, 2,2 dimethoxyethane and 2-butanone over the cultures of AGS, SNU-1, GES-1 cells and medium.

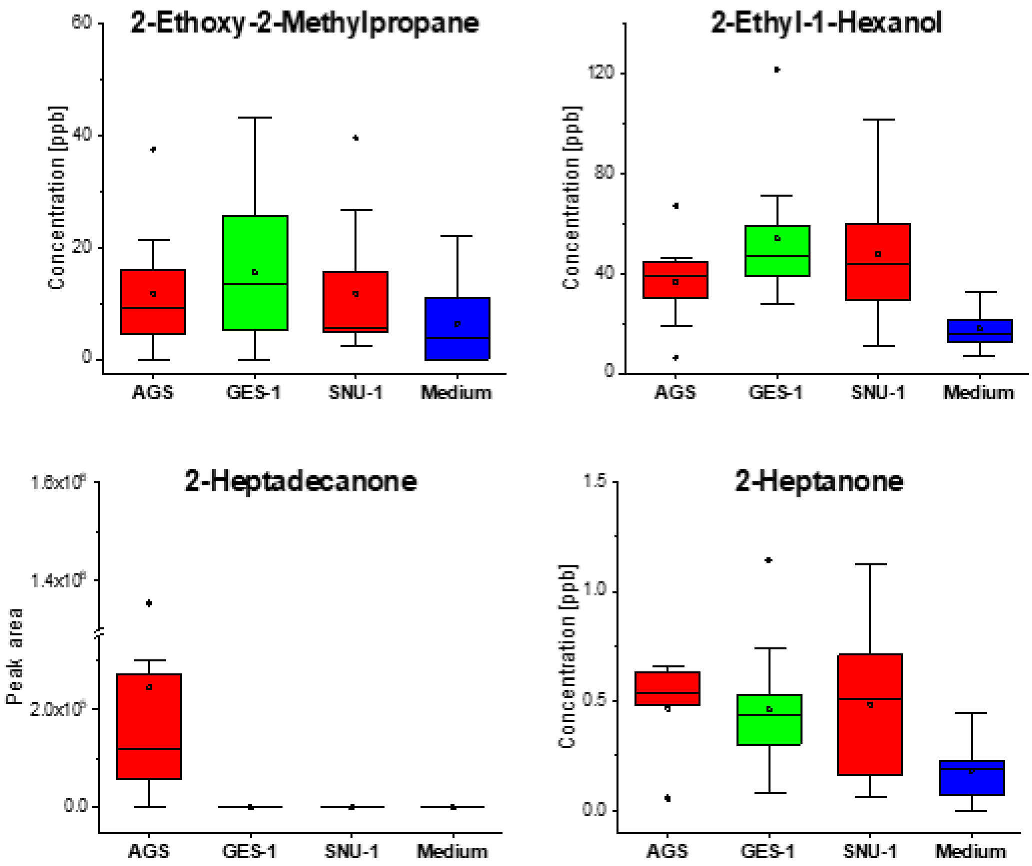

Figure S2. Comparison of the headspace concentrations of 2-ethoxy-2-methylpropane, 2-ethyl-1-hexanol, 2-heptadecanone and 2-heptanone over the cultures of AGS, SNU-1, GES-1 cells and medium.

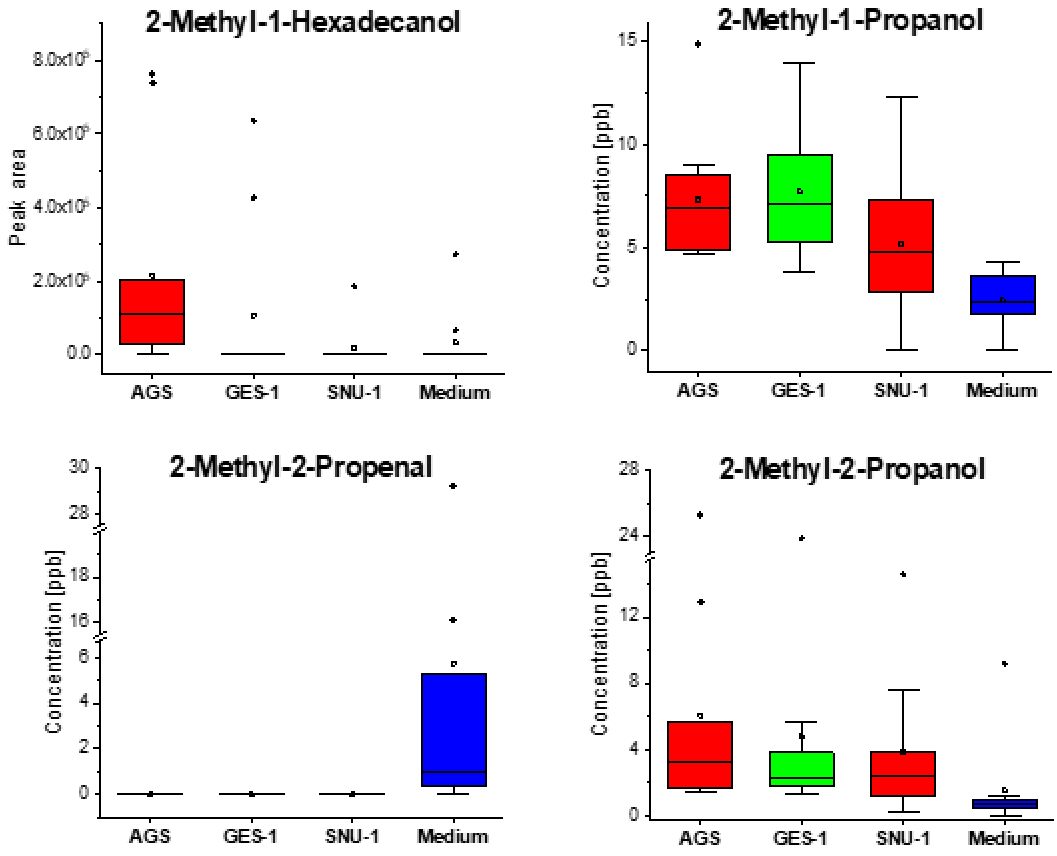

Figure S3. Comparison of the headspace concentrations of 2-methyl-1-hexadecanol, 2-methyl-2-propenal, 2-methyl-1-propanol and 2-methyl-2-propanol over the cultures of AGS, SNU-1, GES-1 cells and medium.

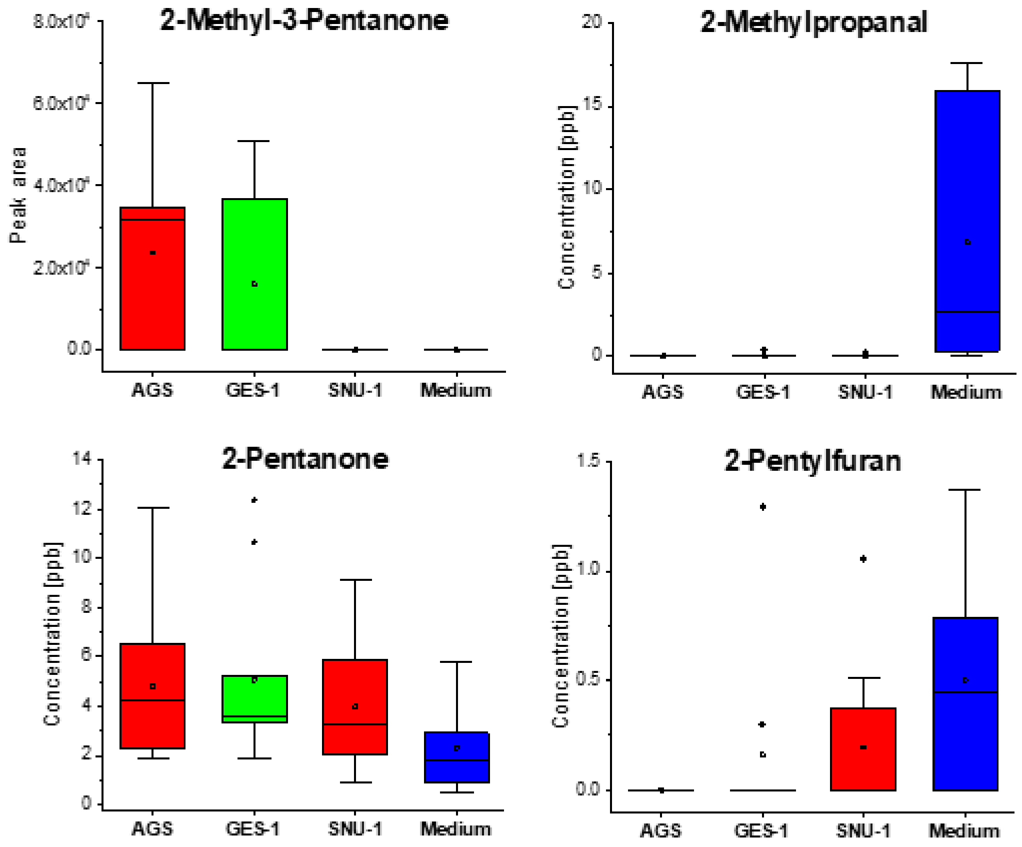

Figure S4. Comparison of the headspace concentrations of 2-methyl-3-pentanone, 2-methylpropanal, 2-pentanone and 2-pentylfuran over the cultures of AGS, SNU-1, GES-1 cells and medium.

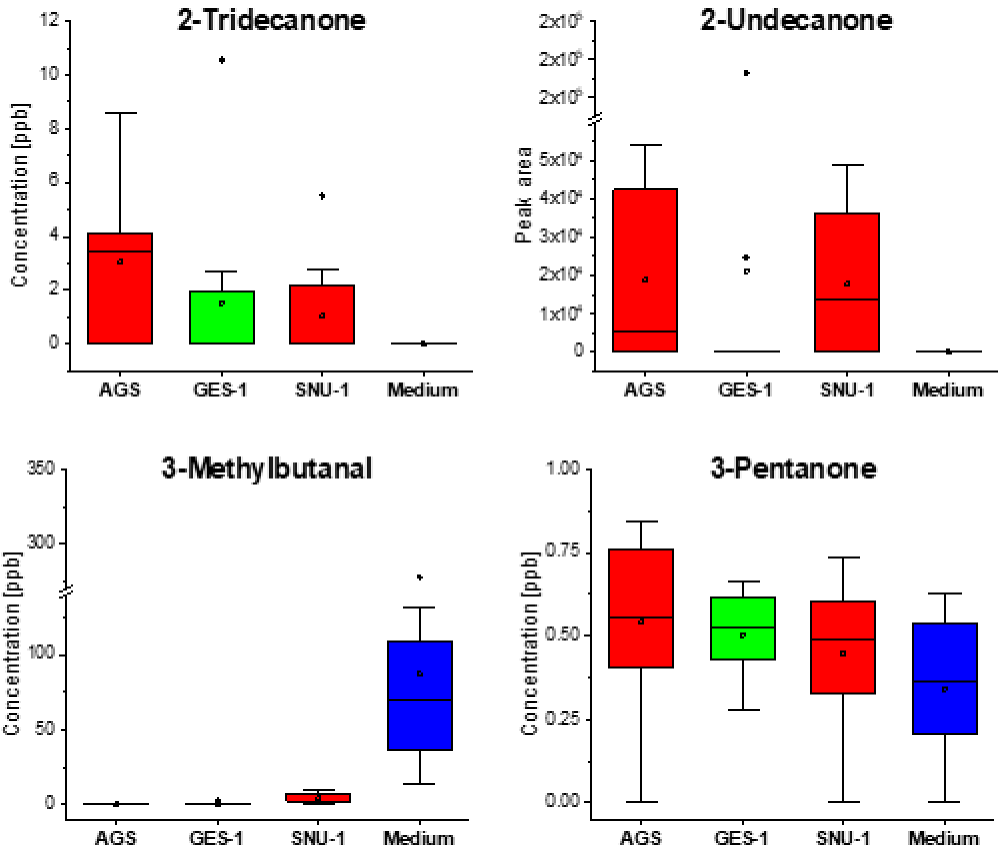

Figure S5. Comparison of the headspace concentrations of 2-tridecanone, 2-undecanone, 3-methylbutanal and 3-pentanone over the cultures of AGS, SNU-1, GES-1 cells and medium.

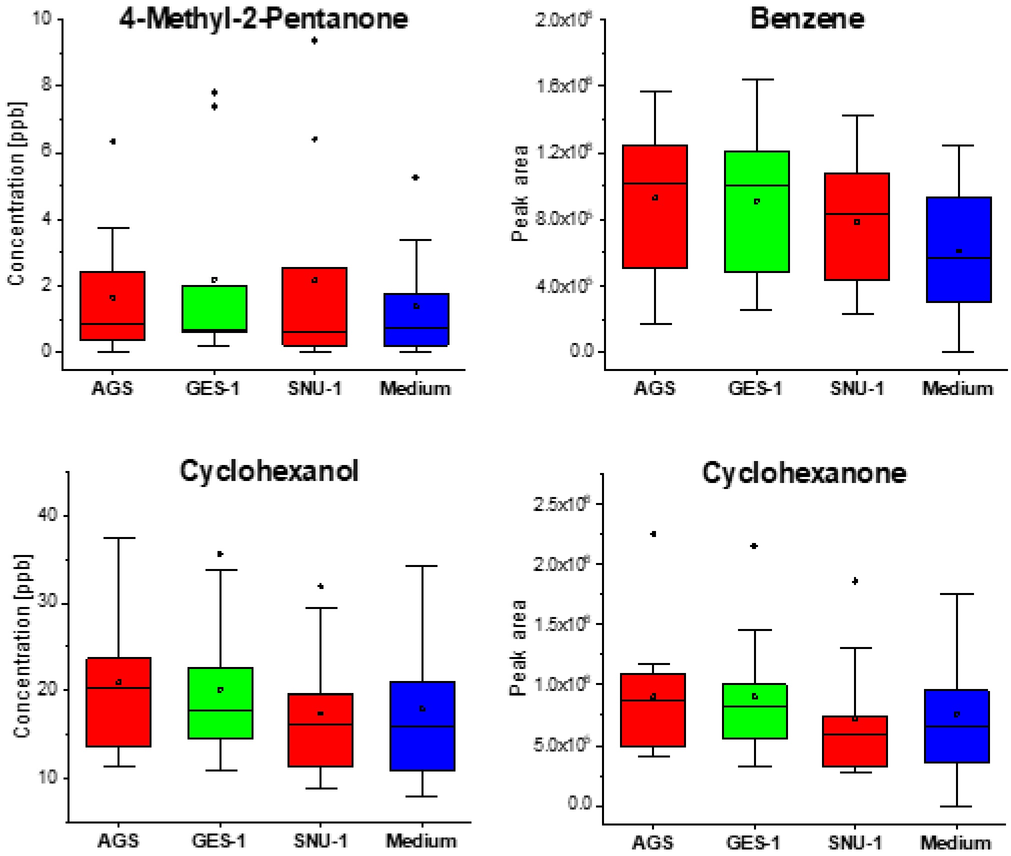

Figure S6. Comparison of the headspace concentrations of 4-methyl-2-pentanone, benzene, cyclohexanol and cyclohexanone over the cultures of AGS, SNU-1, GES-1 cells and medium.

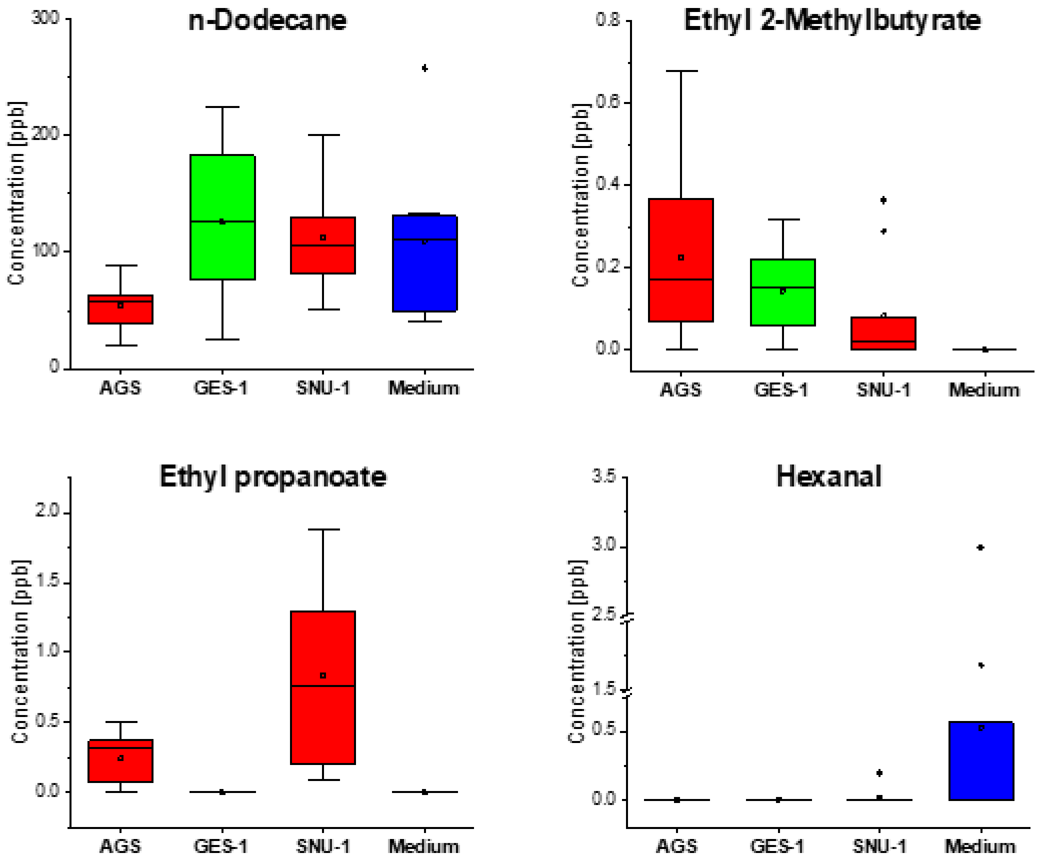

Figure S7. Comparison of the headspace concentrations of n-dodecane, ethyl 2-methylbutyrate, ethyl propanoate and hexanal over the cultures of AGS, SNU-1, GES-1 cells and medium.

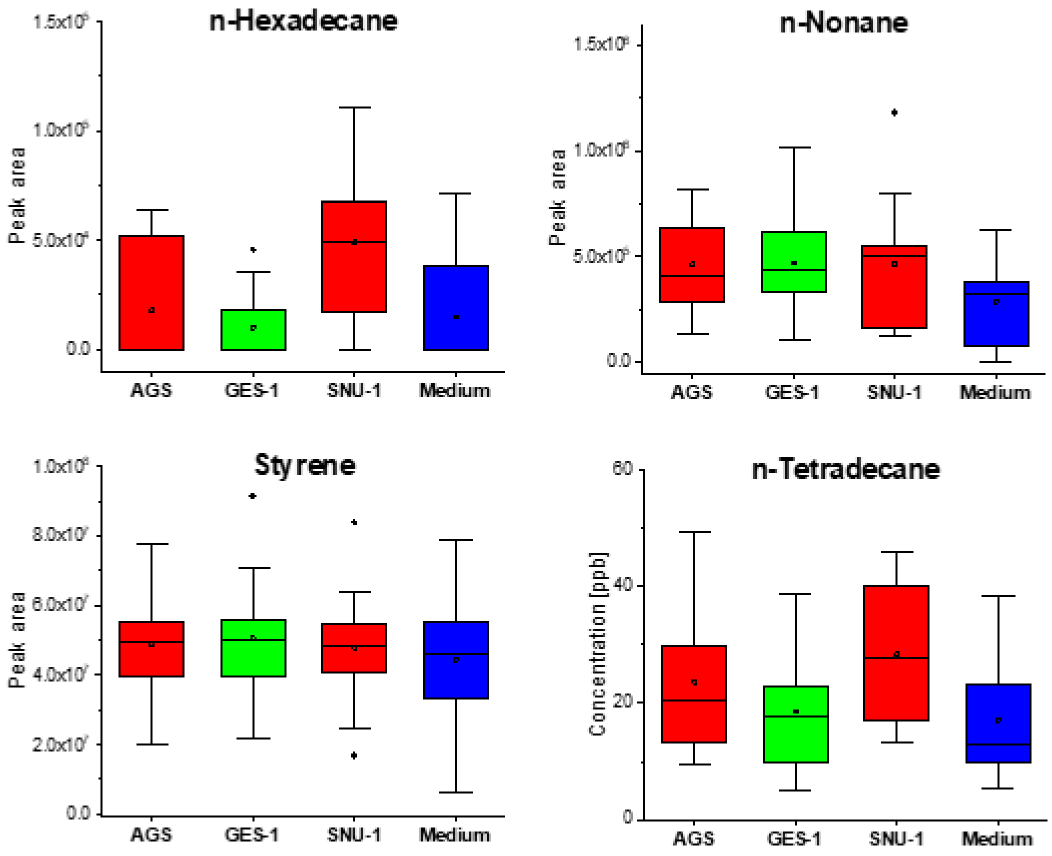

Figure S8. Comparison of the headspace concentrations of n-hexadecane, n-nonane, styrene and n-tetradecane over the cultures of AGS, SNU-1, GES-1 cells and medium.

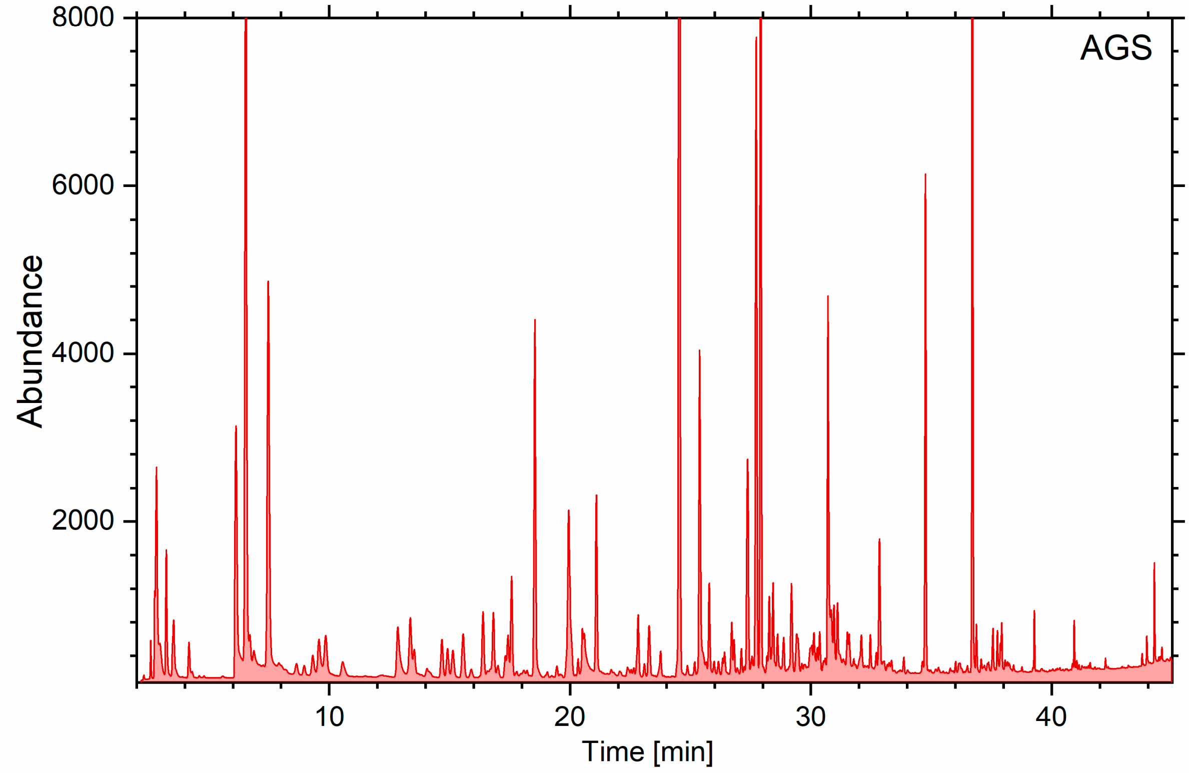

Figure S9 Exemplary chromatogram from HS-NTE-GCMS analysis of an AGS cell culture head-space.

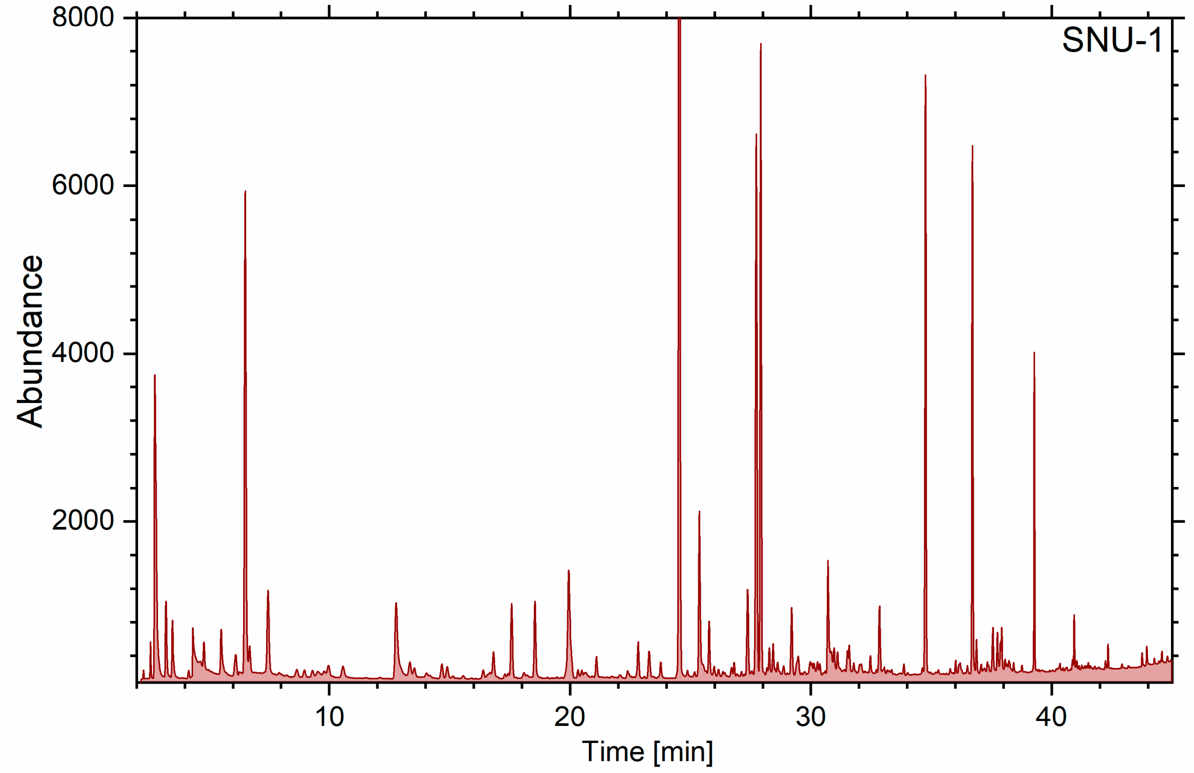

Figure S10 Exemplary chromatogram from HS-NTE-GCMS analysis of a SNU-1 cell culture head-space.

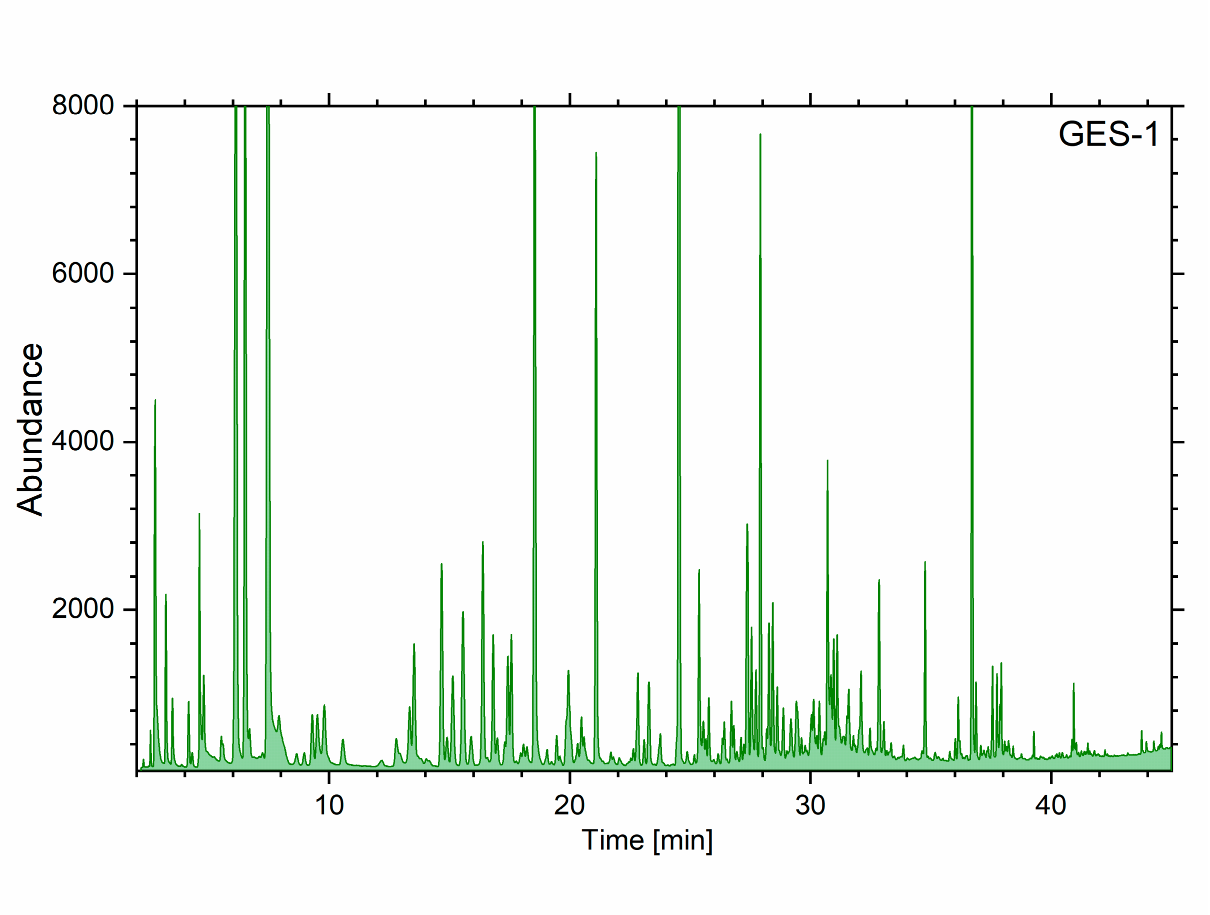

Figure S11 Exemplary chromatogram from HS-NTE-GCMS analysis of a GES-1 cell culture head-space.

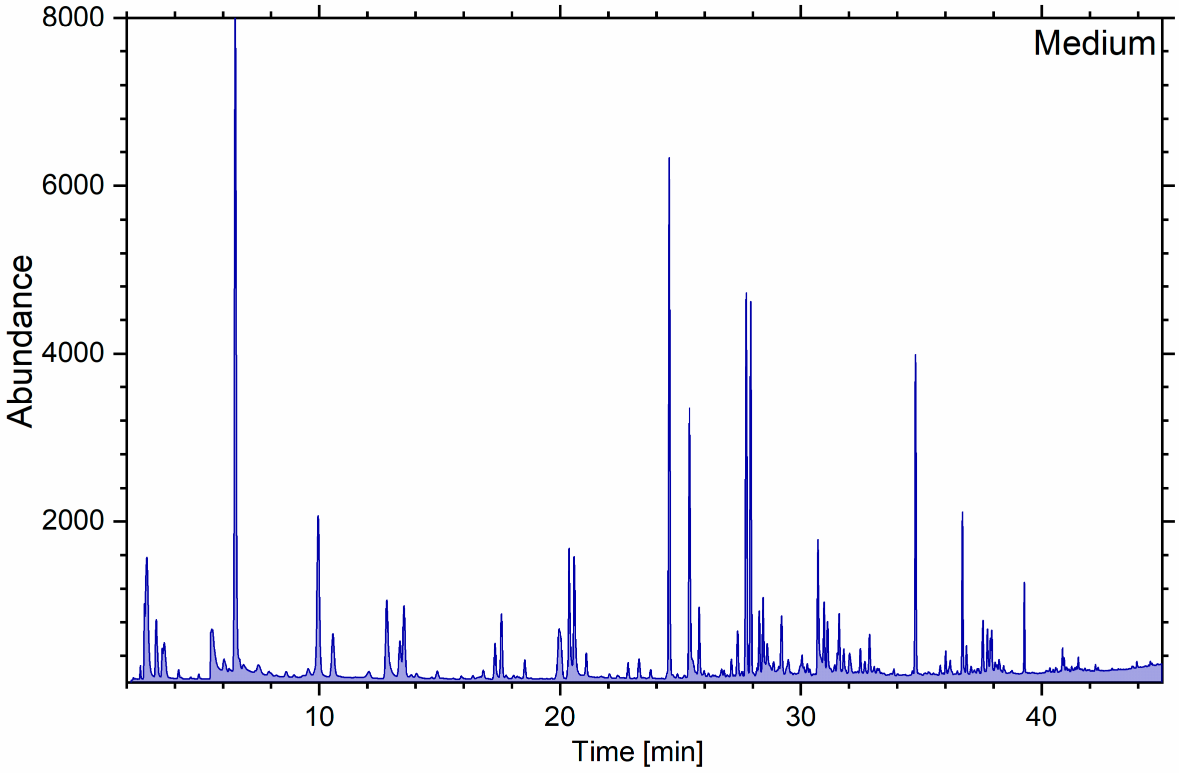

Figure S12 Exemplary chromatogram from HS-NTE-GCMS analysis of a medium head-space.
